# Multi-Type Ship Detection in Complex Marine Backgrounds Using an Enhanced YOLO-Based Network

**DOI:** 10.3390/s26092718

**Published:** 2026-04-28

**Authors:** Anran Du, Huiqi Xu, Wenqiang Yao

**Affiliations:** Naval Aviation University, Yantai 264001, China; xdly0312@163.com (A.D.); 15615750842@163.com (W.Y.)

**Keywords:** YOLOv13, ship detection, feature fusion, attention mechanism, loss function

## Abstract

**Highlights:**

**What are the main findings?**

- A novel AK-DSAM-YOLOv13 detector is proposed, integrating three complementary modules: AKC3k2 for adaptive multi-scale feature extraction via alterable kernel convolution, DyDSAM for dynamic upsampling with dual-stream attention to suppress background clutter, and AIoU loss for precise bounding box regression through joint optimization of overlap area, center distance, and aspect ratio.
- Extensive experiments on the self-built CM-Ships dataset and public SeaShips and McShips benchmarks demonstrate that AK-DSAM-YOLOv13 achieves superior detection accuracy and generalization—with 88.6% precision and 87.7% mAP50 on CM-Ships, and 95.1 % and 93.8% mAP50 on SeaShips and McShips, respectively—while maintaining real-time inference speed and low computational overhead.

**What are the implications of the main findings?**

- The proposed algorithm provides an efficient and reliable solution for intelligent maritime visual monitoring in complex environments, effectively addressing the challenges of small-target missed detection, feature discrimination difficulty, and inaccurate localization under adverse weather, cluttered backgrounds, and dense vessel arrangements.
- The modular design of AKC3k2, DyDSAM, and AIoU-Loss offers a flexible architectural paradigm that can be adapted to other real-time object detection tasks requiring robustness to scale variation, occlusion, and background interference, such as autonomous driving and industrial inspection.

**Abstract:**

Accurate detection of ship targets in complex marine environments is fundamental to ensuring maritime security and safeguarding maritime rights. With the increasing diversity of vessel types and configurations, achieving precise identification of multiple ship classes amidst dynamic interference and cluttered backgrounds has emerged as a formidable challenge in marine surveillance. To address three pervasive issues in ship target detection—namely, high false-negative rates for small targets, inadequate feature discrimination, and imprecise localization—this paper proposes AK-DSAM-YOLOv13, a multi-scale detection algorithm specifically tailored for complex marine scenarios. Built upon the YOLOv13n architecture, the proposed algorithm implements integrated optimizations across the backbone network, neck structure, and loss function. First, a lightweight cross-scale feature extraction module, AKC3k2, is constructed by incorporating Alterable Kernel Convolutions (AKConv) to reconstruct the feature extraction path, thereby significantly enhancing the representation of multi-scale targets. Second, a Dynamic Up-Sampling Dual-Stream Attention Merging (DyDSAM) structure is designed, which integrates the DySample operator with a Dual-Stream Attention Mechanism (DSAM) to effectively suppress background clutter and improve feature fusion accuracy. Third, an Accuracy-Intersection-over-Union (AIoU) loss function is introduced to jointly optimize overlap area, center distance, and aspect ratio, enhancing localization robustness for small-scale objects. Experimental results on the self-built CM-Ships dataset, as well as the public SeaShips and McShips datasets, demonstrate that AK-DSAM-YOLOv13 significantly outperforms baseline models in detection accuracy, recall, and generalization capability while maintaining a low computational overhead. This research provides an efficient and reliable technical framework for intelligent maritime visual monitoring in complex environments.

## 1. Introduction

With the rapid expansion of the marine economy, the imperative to safeguard maritime rights and interests has intensified, rendering the construction of robust maritime security defense systems increasingly critical. Accurate and efficient detection of maritime vessels has emerged as a foundational task in domains such as maritime traffic surveillance and maritime defensive situational awareness [1]. Traditional object detection algorithms typically follow a three-stage pipeline: candidate region selection, feature extraction, and classification. These methods rely on handcrafted features—such as SIFT and HOG—and classifiers like SVM to represent and distinguish targets [2]. However, the efficacy of such approaches is heavily contingent upon heuristic expertise, and their feature representation capabilities remain inherently limited. Under the dynamic interference characteristic of complex marine environments, their generalization performance tends to deteriorate sharply, rendering them inadequate for practical deployment.

In recent years, object detection frameworks based on Convolutional Neural Networks (CNNs) have demonstrated superior performance and robustness in complex scenarios, leveraging adaptive hierarchical learning and end-to-end joint optimization. Mainstream algorithms are generally categorized into two-stage detectors, exemplified by the R-CNN series [3], and single-stage detectors, including the SSD, RetinaNet, and YOLO series [4]. Among these, the YOLO series has been extensively applied to various detection tasks involving ships, vehicles, and aircraft due to its streamlined architecture and optimal trade-off between accuracy and inference speed, thereby becoming the de facto baseline for ship detection. To meet the practical requirements of ship detection under challenging sea conditions, numerous enhanced models based on the YOLO framework have emerged. For instance, Reference [5] proposes ADV-YOLO, an improved SAR ship detection model based on YOLOv8; by incorporating the Spatial-to-Depth (SPD) module, the Deep Weighted Residual (DWR) module, and the Weighted Intersection over Union (WIoU) loss, it significantly improves feature extraction for small-scale and multi-scale vessels, albeit at the cost of increased model complexity and computational demand. Reference [6] introduces a Deep Weighted Feature Pyramid Network (DWFPN) utilizing dilated convolutions and attention mechanisms to achieve cross-level weighted feature fusion, thereby mitigating feature attenuation for small targets; however, it remains susceptible to feature confusion in dense berthing scenarios. Reference [7] develops an Edge-Informative Infrared Ship Detection network (EGISD-YOLO), which enhances feature reusability through a Dense-Cross-Stage Partial (Dense-CSP) structure and integrates shallow edge cues with deep semantic features via edge-guided fusion. While this improves localization, edge features remain highly sensitive to noise interference from sea clutter and atmospheric turbulence. Recent research has also focused on enhancing detection robustness under degraded illumination conditions. For instance, Reference [8] proposes an illumination-invariant hierarchical feature enhancement network for low-light object detection, complementing our work which emphasizes multi-scale feature extraction and background suppression under diverse complex marine conditions.

Despite significant progress in specific scenarios, existing research still faces limitations regarding robustness in complex marine environments, the equilibrium between accuracy and speed, and adaptability for lightweight deployment. Considering the prospective demands of maritime monitoring, current ship detection methods primarily confront the following challenges: (1) Detection performance degrades significantly under variable conditions such as adverse weather, terrestrial background interference, target overlapping, and partial occlusion; (2) small coastal vessels exhibit a low pixel footprint and weak feature saliency, making them prone to submersion within sea clutter and background noise; (3) in constrained areas like ports, dense vessel distributions lead to severe mutual occlusion, frequently resulting in missed detections and boundary ambiguity.

To address these challenges, this paper proposes the AK-DSAM-YOLOv13 detection model, an enhancement of the baseline YOLOv13n architecture. Its primary contributions are as follows:

1. Development of a Lightweight Cross-Scale Feature Extraction Module (AKC3k2): By integrating Alterable Kernel Convolution (AKConv) into the DS-C3k2 structure within the backbone and neck networks to replace original Bottleneck units, we construct the AKC3k2 module with an adaptive receptive field. This enhances the model’s capacity for multi-scale feature representation and contextual modeling.
2. Design of a Dynamic Up-Sampling Dual-Stream Attention Merging (DyDSAM) Structure: The dynamic up-sampling operator, DySample, is introduced into the neck network to replace conventional interpolation methods. Coupled with the Dual-Stream Attention Module (DSAM), this architecture suppresses information loss during up-sampling while improving cross-level feature alignment and fusion precision.
3. Proposal of an Accuracy-Intersection-over-Union (AIoU) Loss Function: The AIoU loss replaces conventional bounding box regression losses by jointly optimizing overlap area, central point distance, and aspect ratio compatibility. This enhances the model’s sensitivity to geometric constraints, thereby improving localization robustness for small targets and dense maritime scenes.

The proposed AK-DSAM-YOLOv13 model is validated through comprehensive comparative and ablation experiments on a visible-light ship dataset that encompasses diverse sea conditions and scale distributions, demonstrating significant advantages in detection accuracy, robustness, and real-time performance.

## 2. Materials and Methods

### 2.1. Overview of the Baseline Model YOLOv13

**Figure 1:**
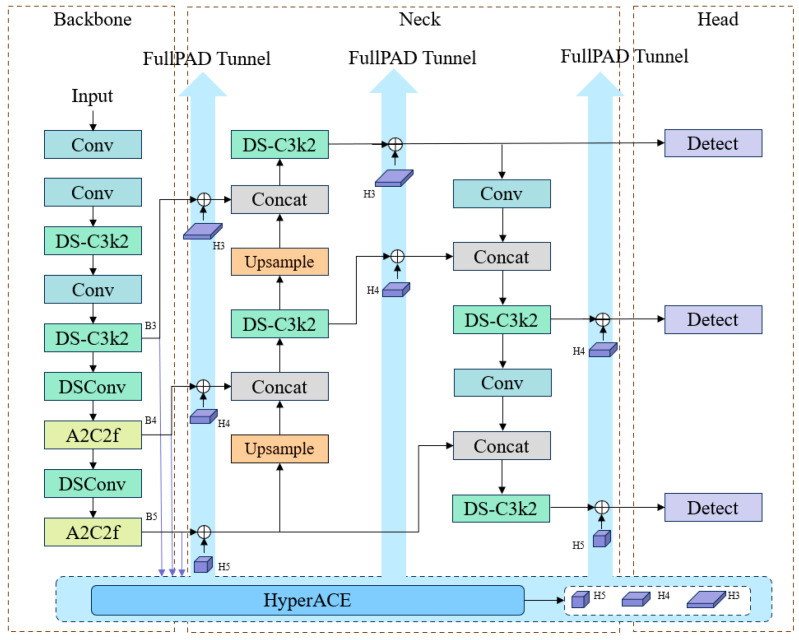
Architecture of YOLOv13 network. Arrows indicate data flow; dashed boxes divide the network into Backbone, Neck, and Head. The ⊕ symbol denotes element-wise addition. Colored rectangles distinguish functional modules: light blue for standard convolution, green for depthwise separable convolution (DS-C3k2, DSConv), yellow for A2C2f, gray for feature fusion and upsampling, purple for detection heads. Blue-highlighted regions mark key innovations: FullPAD Tunnel for global feature transmission and HyperACE for multi-scale feature enhancement. The backbone adopts CSPDarknet with cross-stage partial connections; the neck uses PANet for bidirectional multi-scale fusion; the detection head employs a decoupled structure for separate classification and regression outputs.

YOLOv13 [9] was officially released on 26 June 2025, through a collaborative effort involving Tsinghua University, Taiyuan University of Technology, Beijing Institute of Technology, and other prominent institutions. As the latest iteration in the YOLO series, its primary objective is to resolve robustness bottlenecks in object detection within highly complex environments. A cornerstone of YOLOv13 is the introduction of the Hypergraph-based Adaptive Correlation Enhancement (HyperACE) mechanism, which adaptively excavates latent high-order correlations to facilitate efficient global feature fusion and cross-scale enhancement. Building upon HyperACE, the architecture implements a Full-Pipeline Aggregation-and-Distribution (FullPAD) paradigm. This mechanism systematically distributes enhanced correlated features across the entire network pipeline, enabling fine-grained information flow and collaborative feature representation. Furthermore, YOLOv13 incorporates Depthwise Separable Convolutions (DSConv), which significantly mitigates model parameterization and computational complexity while maintaining high-fidelity performance. The detailed network architecture is illustrated in Figure 1.

While YOLOv13 demonstrates commendable performance in generalized object detection tasks, its efficacy remains constrained in complex maritime environments. Specifically, the model exhibits high susceptibility to performance degradation when encountering variable meteorological conditions, intricate background interference, and target occlusion [9,10]. For small-scale vessels in coastal proximity, the architecture suffers from significantly elevated false-negative and false-positive rates, primarily due to its insufficient sensitivity to low-contrast features within visible-light imagery [11]. In this study, we adopt the lightweight YOLOv13n variant as the baseline model. All quantitative experiments and qualitative comparisons are based on YOLOv13n unless otherwise specified.

### 2.2. Overall Architecture of the Proposed AK-DSAM-YOLOv13

**Figure 2:**
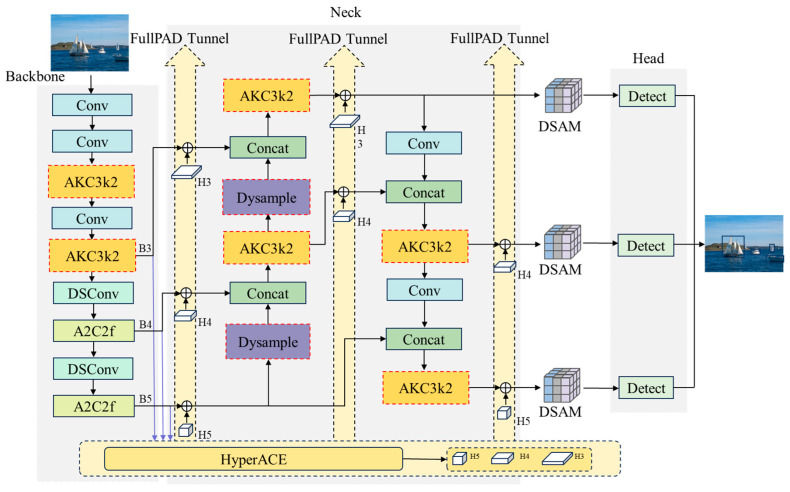
Architecture of AK-DSAM-YOLOv13 network. The network integrates three core improvements into the YOLOv13n baseline: the AKC3k2 feature extraction module (backbone), which replaces the original DS-C3k2 Bottleneck with AKConv to achieve adaptive receptive fields; the DyDSAM neck structure, consisting of DySample dynamic upsampling and the Dual-Stream Attention Module (DSAM), which enhances feature fusion accuracy and suppresses background interference; and the AIoU regression loss at the detection head, which optimizes bounding box regression. Different colors and arrow styles indicate different modules and data flows; dashed boxes highlight the replaced components.

To surmount these challenges, this paper proposes AK-DSAM-YOLOv13, an enhanced detection framework. By refining convolutional modules, optimizing the feature fusion architecture, integrating a dual-stream attention mechanism, and formulating a more robust loss function, the proposed model significantly bolsters detection precision and robustness for multi-scale maritime targets. The comprehensive network architecture is illustrated in Figure 2.

### 2.3. Lightweight Cross-Scale Feature Extraction Module Based on Variable-Kernel Convolution

The baseline YOLOv13n employs the DS-C3k2 module for feature extraction, which leverages Depthwise Separable Convolutions (DSConv) to reduce parameter redundancy and computational overhead while preserving feature representation through parallel branching. However, this architecture exhibits inherent limitations; specifically, the sequential execution of depthwise and pointwise convolutions precludes effective cross-channel interactions [12,13]. Consequently, this constraint restricts the model’s capacity to capture high-order feature correlations and global contextual information, thereby hindering its performance in complex scenarios and small-object detection tasks.

**Figure 3:**
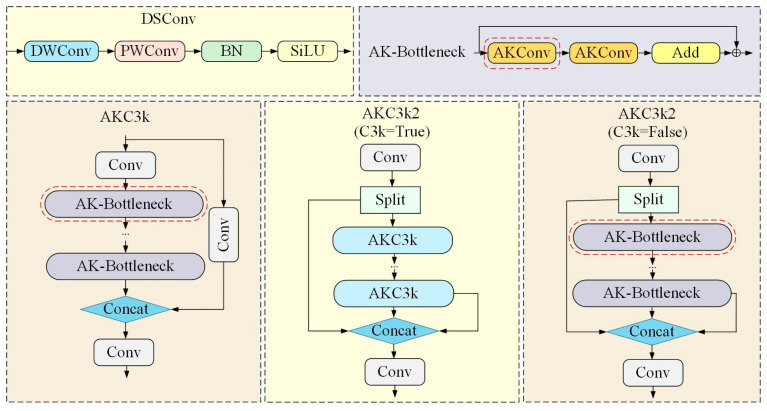
Architectural diagram of the proposed AKC3k2 module. Arrows indicate the direction of data flow, and the ⊕ symbol denotes element-wise addition. Different background colors distinguish different functional components: light yellow for basic convolution operators, light purple for the AK-Bottleneck block, light beige for the overall AKC3k/AKC3k2 modules, and light blue for the nested AKC3k submodules.The module inherits the cross-stage partial connection architecture of C3k2, but replaces all DS-C3k2 Bottleneck blocks with AK-Bottleneck blocks (highlighted with red dashed boxes). Each AK-Bottleneck uses AKConv layers instead of depthwise separable convolution, introducing a learnable offset prediction branch for adaptive receptive fields.

To enhance cross-scale feature extraction, we introduce Alterable Kernel Convolution (AKConv) [14] and construct a novel AKC3k2 module, which replaces the standard Bottleneck blocks in the original DS-C3k2 architecture. The architecture of the improved module is depicted in Figure 3.

In our default configuration (used in shallow backbone stages and the entire neck), each AKC3k2 module contains two AK-Bottleneck blocks (c3k = False). In each AK-Bottleneck, a single AKConv layer (kernel size 5) replaces the depthwise separable convolution of the original DS-C3k2 Bottleneck. For deep backbone stages (Stage3, Stage4), we use a variant with c3k = True, where each AKC3k2 module contains a stack of multiple AK-Bottleneck blocks (i.e., an AKC3k submodule) to enhance semantic feature extraction. All Bottleneck blocks in the DS-C3k2 modules of both the backbone and the neck are replaced with AK-Bottleneck.

**Figure 4:**
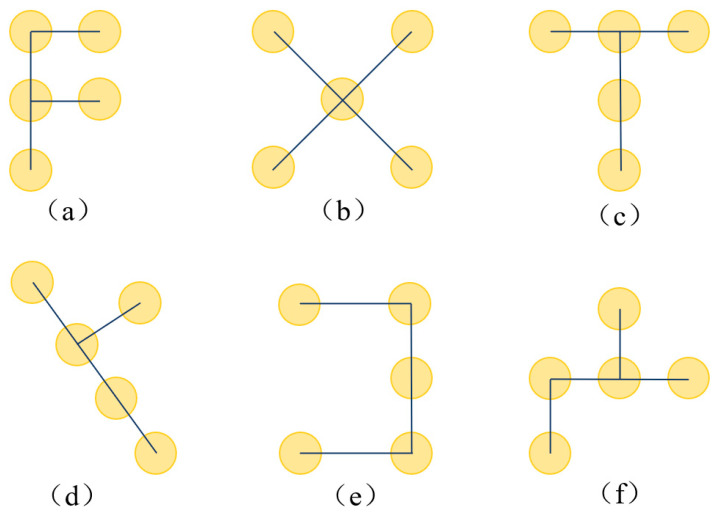
Example of AKConv sampling layout with kernel size 5. The illustration shows six representative sampling point distributions that AKConv can generate, combining regular grids with learned offsets. (**a**) F-shaped asymmetric rectangular sampling; (**b**) X-shaped diagonal cross sampling; (**c**) T-shaped orthogonal cross sampling; (**d**) Oblique Y-shaped non-orthogonal branch sampling; (**e**) C-shaped edge contour sampling; (**f**) Inverted T-shaped corner cross sampling. These patterns are representative examples tailored for ship detection tasks.

AKConv implements a dynamically adjustable sampling mechanism. Unlike standard convolutions with fixed sampling grids, AKConv first generates an initial sampling coordinate set that integrates both regular and irregular distributions, establishing a foundation for arbitrary-shape feature sampling. Figure 4 illustrates an example of the initial sampling configuration for a kernel size of 5. Although the illustrated configuration is selected for demonstration purposes, AKConv is not restricted to this pattern; it possesses the capability to generate arbitrary sampling shapes adapted for various applications.

Subsequently, a lightweight convolutional layer predicts learned offsets to dynamically refine the sampling point positions, enabling the kernel to adaptively align with salient feature regions based on localized image content. This mechanism empowers the model to optimize its sampling strategy for diverse object morphologies during the training phase, significantly strengthening its representational capacity for deformable and multi-scale maritime targets.

To provide a theoretical foundation for the efficiency of AKConv, we analyze its parameter complexity. For a standard convolution with a kernel size of k×k, the parameter count is $C_{in}\times k^{2}\times C_{out}$, scaling quadratically with k Deformable Convolution (DCN) introduces an offset prediction layer with $2k^{2}$ output channels, leading to $C_{in}\times k^{2}\times 2k^{2}=2C_{in}k^{4}$ additional parameters—a quartic growth. In contrast, AKConv decouples offset prediction from the kernel size. A lightweight predictor generates approximately $2k^{2}$ offset parameters shared across spatial positions, yielding a total parameter count of $C_{in}\times k^{2}\times C_{out}+O(k^{2})$. Thus, the dominant term remains $O(k^{2})$, ensuring that AKConv’s parameter count scales linearly with the kernel size, preserving feature extraction flexibility while enabling lightweight deployment.

To clarify the design evolution, we compare the proposed AKC3k2 with the original DS-C3k2. Table 1 summarizes the quantitative differences in module structure, convolution operation, receptive field, parameter count, and computational cost. The parameters and computational costs are calculated under a unified setting (input feature map of 256×256×128, base kernel size of 5×5). 

To further evaluate the effectiveness of AKC3k2 on small-scale ship targets, we conducted an ablation study utilizing a single-module replacement on the CM-Ships small-scale subset (object area < $32^{2}$ pixels). The results are presented in Section 3.3.

**Figure 5:**
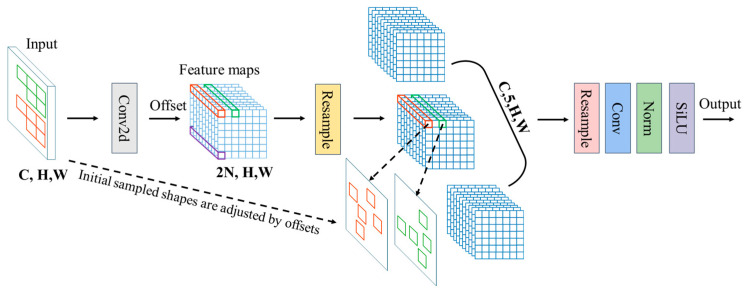
Architecture of AKConv. Arrows indicate data flow; dashed lines show how learned offsets adjust initial sampling shapes. Variable definitions: C = input channels, H = height, W = width, N = number of sampling points per spatial position (here N = 5, so 2N corresponds to x and y offsets). Different colored rectangles represent network layers (gray for 2D convolution, yellow/pink for resampling, blue for convolution, green for batch normalization, purple for SiLU activation). Red and green squares denote two groups of adaptively learned sampling points. AKConv dynamically adjusts sampling positions via learned offsets, enabling content-adaptive feature alignment.

Compared to alternative convolutional operators—such as Partial Convolution (PConv) [15], Multi-Branch Re-param Convolution (MBRConv) [16], and Pinwheel-shaped Convolution [17]—AKConv exhibits more targeted adaptability specifically tailored for ship detection. To balance detection accuracy, parameter efficiency, and computational cost, we select a kernel size of 5 for the AKConv used in our AKC3k2 module. This choice is based on the general performance trend observed on standard benchmarks (e.g., COCO2017), where k = 5 achieves a competitive mAP50 while maintaining a lightweight footprint, aligning with the real-time deployment requirements of maritime monitoring systems. The comprehensive structural design of AKConv is illustrated in Figure 5.

Furthermore, the adaptive receptive field of AKConv dynamically adjusts to multi-scale vessel geometries, effectively capturing features of both large cargo ships and small fishing boats. Moreover, its linear parameter growth with kernel size ensures lightweight deployment, satisfying the real-time processing requirements of maritime monitoring systems.

### 2.4. Dynamic Up-Sampling Dual-Stream Attention Fusion Feature Structure

#### 2.4.1. Neck Dynamic Up-Sampling Module

To enhance feature map resolution and bolster detail retention, researchers have recently introduced various content-aware dynamic upsampling operators. For instance, CARAFE [18] utilizes subnetworks to generate dynamic convolutional kernels for feature reorganization; FADE [19] aggregates multi-resolution features to produce structure-aware dynamic kernels; and SAPA [20] constructs awareness kernels by calculating the affinity between high- and low-resolution features. Despite their efficacy, these approaches often incur substantial computational complexity and implementation overhead. Furthermore, certain operators necessitate high-resolution guidance, thereby restricting their deployment in lightweight detection frameworks.

To optimize the feature upscaling performance of the Neck Network and preserve the structural integrity of small-scale maritime targets, this study integrates the lightweight and efficient dynamic upsampling operator, DySample [21], to replace the conventional upscaling modules. Centered on the principle of dynamic point sampling, DySample implements feature resampling via an adaptive sampling set. Unlike rigid sampling rules such as bilinear or bicubic interpolation, which apply uniform sampling across spatial positions and inevitably introduce aliasing artifacts or edge blurring at object boundaries, DySample learns content-aware offsets that align sampling points with salient structures. For ship detection, vessel boundaries frequently include slender protrusions such as masts and bows, as well as narrow gaps between berthed ships. Preserving these edge details during upsampling therefore supports sharper localization and better separation between adjacent targets.

**Figure 6:**
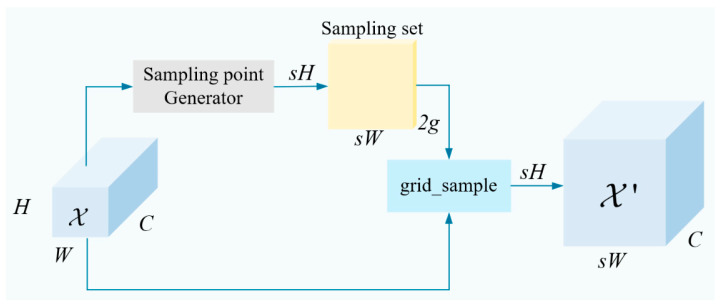
Core workflow of the DySample dynamic upsampling operator. Variable definitions: H = input height, W = input width, C = input channels, sH = output height, sW = output width, g = number of groups for sampling point generation, 2g = offset channels (x and y coordinates for each group). “grid_sample” denotes the grid sampling function. The input feature map X is resampled using a content-aware sampling set S learned by a lightweight generator, producing the upsampled output $X^{\prime }$.

In contrast to dynamic convolution-based upsamplers such as CARAFE, which require kernel generation and often rely on high-resolution guidance, DySample eliminates kernel generation entirely. Instead, it predicts spatial offsets via a lightweight linear layer followed by pixel shuffling, maintaining low memory usage and low inference latency. This efficiency is critical for real-time maritime surveillance, where processing must sustain continuous frame rates from shore-based or shipboard cameras. The core workflow of DySample is illustrated in Figure 6.

Given an input feature map X of size $C\times H_{1}\times W_{1}$ and a sampling set S of size $2\times H_{2}\times W_{2}$ (where the first dimension of size 2 represents the x and y coordinates), the grid sampling function uses the sampling coordinates specified in S to resample X, generating an output feature map $X^{\prime }$ of size $C\times H_{2}\times W_{2}$. This process can be expressed as:

(1) $$X^{\prime }=\;grid\_sample\left(X,S\right)$$

Let the upsampling scale factor be s and the input feature map X be of size C×H×W. First, a linear layer is applied to X, where the number of input and output channels are C and $2s^{2}$, respectively, generating an offset O with dimensions $2s^{2}\times H\times W$. This offset is then reshaped to 2×sH×sW via Pixel Shuffling [22]. Finally, the sampling set S can be obtained by adding the offset O to the original sampling grid G:

(2) S = G+linear(X) 

**Figure 7:**
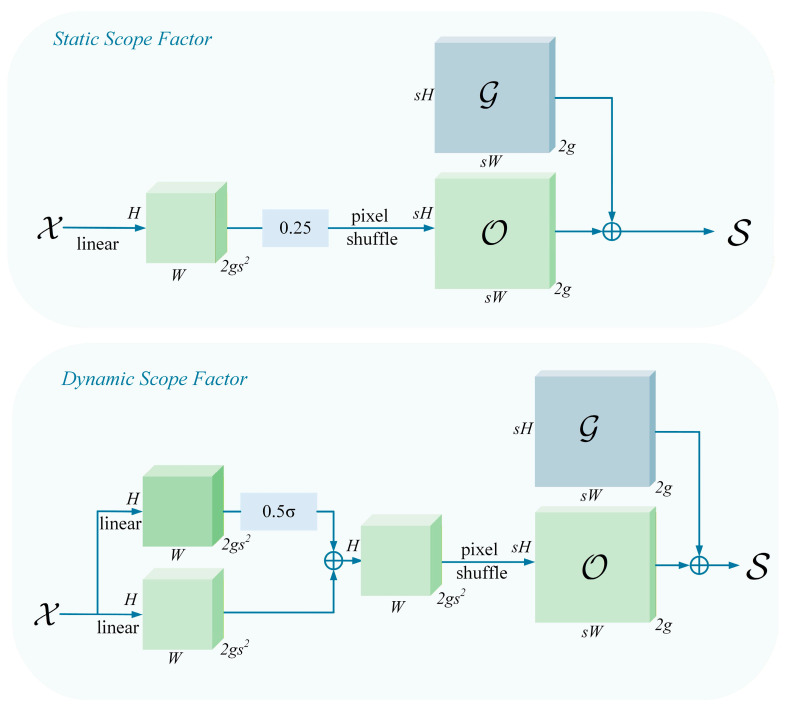
Sampling point generator in DySample. Variable definitions: X = input feature map, H = height of the input feature map, W = width of the input feature map, s = upsampling scale factor, sH = height of the upsampled feature map, sW = width of the upsampled feature map, g = number of groups for sampling point generation, 2g = total output channels for x and y coordinate offsets, G = static scope factor tensor, O = learned offset tensor, S = final generated sampling set. The static offset 0.25 and the dynamic range factor 0.5σ follow empirical settings from the original DySample implementation. The former ensures non-overlapping sampling points for 2× upscaling, while the latter serves as the default dynamic range.

To enhance the adaptability of the sampling process, DySample further incorporates a dynamic range factor mechanism. As illustrated in Figure 7, this mechanism employs an additional small subnetwork to predict the range factor. After normalization via a sigmoid function, it modulates the offset to achieve dynamic control over the sampling range, thereby further improving the quality and stability of the upscaled features.

By learning content-aware offsets, DySample preserves sharp vessel boundaries and alleviates edge blurring caused by traditional interpolation. This property is crucial for distinguishing closely arranged ships in dense berthing scenarios and for accurately localizing small-scale or distant targets with limited edge information.

#### 2.4.2. Neck Attention Module

To facilitate the integration of multi-scale semantic and granular detail and to bolster the model’s discriminative capacity between foreground targets and complex sea surface backgrounds, this study adapts the Dual-Stream Attention Module (DSAM) [23] from the Boundary-Aware Feature Fusion Network (BAFNet). The module is strategically embedded into the feature fusion path of the YOLOv13n architecture.

**Figure 8:**
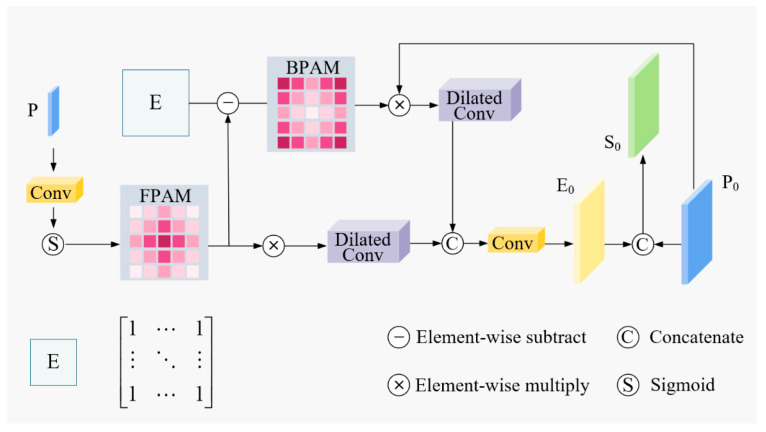
Architecture of the DSAM module. Arrows indicate data flow. Colored rectangles distinguish functional components: light blue for input feature maps, yellow for convolution operations and intermediate features, gray for attention modules and dilated convolutions, green for spatial features, dark blue for the final output. The DSAM employs parallel foreground and background attention streams: FPAM selectively amplifies target-related regions, while BPAM suppresses interference from non-target elements such as sea clutter and coastal structures. The two streams are fused with low-level spatial features and processed through multi-scale dilated convolutions to capture contextual information.

The fundamental premise of DSAM is to explicitly characterize the complementary synergy between the target and its local environment through parallel foreground and background attention streams. This mechanism adaptively amplifies the feature response of maritime vessels while concurrently suppressing interference from non-target elements, such as breaking waves, cloud cover, and coastal islands. By integrating the DSAM module, YOLOv13 achieves a more precise saliency focus on vessel targets, reinforcing critical discriminative features, including morphological edges and textural nuances. Consequently, this architecture significantly improves detection robustness and localization precision under fluctuating sea states and heterogeneous backgrounds. The comprehensive architecture of the DSAM module is illustrated in Figure 8.

First, the highest-level semantic features generated by the encoder are leveraged to produce a Foreground Priority Attention Map (FPAM) and a Background Priority Attention Map (BPAM). These maps are designed to selectively amplify target-related regions and suppress background noise, respectively, thereby facilitating more discriminative feature representation. The computational procedure is formulated as follows:

(3) $$\left\{\begin{array}{c} A^{F}=\sigma (U(\mathrm{conv}(P)))\\ A^{B}=E-A^{F} \end{array}\right.$$

where $A^{F}$ denotes the FPAM, $A^{B}$ denotes the BPAM, U(⋅) represents upsampling, conv(⋅) is a convolution operation using a 1×1 kernel, and σ(⋅) is the sigmoid function.

Subsequently, the generated attention maps are fused with the low-level spatial detail features $P_{0}$ through element-wise weighting. Multi-scale dilated convolutions are introduced to capture contextual information under different receptive fields, thereby enhancing the representation capability for small-scale ships:

(4) $$E_{0}=L\left(\left[{⊔}_{i=1}^{4}D_{i}\left(P_{0}⊙A^{F}\right),{⊔}_{i=1}^{4}D_{i}\left(P_{0}⊙A^{B}\right)\right]\right)$$

where ⊙ denotes element-wise multiplication, $D_{i}$ represents a group of multi-scale convolutions (including a 1×1 convolution and dilated convolutions with dilation rates of 3, 5, and 7), and L is the output convolution layer.

Finally, the enhanced features $E_{0}$ are concatenated with the original low-level features $P_{0}$ to obtain the dual-stream attention features that integrate foreground enhancement and background suppression:

(5) $$S_{0}=\left[\begin{array}{c} E_{0},P_{0} \end{array}\right]$$

The resulting feature map $S_{0}$, obtained through the aforementioned dual-stream attention fusion, simultaneously contains enhanced semantic information of foreground targets and suppressed representations of background regions. This feature is subsequently fed into the detection head for localization and classification, significantly improving the model’s discriminative ability and localization accuracy for ship targets, especially small-scale and occluded ones, thereby achieving more accurate and stable ship detection in complex marine backgrounds.

The DSAM module is specifically tailored for ship detection in complex marine environments. By constructing parallel foreground and background attention streams, it adaptively enhances the feature responses of ship targets while suppressing interference from sea clutter, coastal facilities, and island obstacles. Such a design effectively improves the feature discriminability of vessels against heterogeneous maritime backgrounds, especially for small and occluded ships that are easily submerged in background noise.

### 2.5. Regression Loss Module Based on Accurate Intersection-over-Union Ratio

Traditional Intersection-over-Union (IoU) and its variants—such as Complete IoU (CIoU) and Distance IoU (DIoU)—exhibit pronounced deficiencies in maritime small-scale object detection. Specifically, when predicted bounding boxes lack overlap with the ground truth or are subject to inclusion relationships, these metrics fail to provide effective gradients for backpropagation. Furthermore, such loss functions are disproportionately sensitive to minor spatial displacements in small targets, frequently inducing training oscillations and subsequently compromising detection precision. To mitigate these issues, this paper adopts the Accuracy-Intersection-over-Union (AIoU) loss function [24] to optimize the bounding box regression process.

While the baseline YOLOv13n (and its predecessor YOLOv8n) utilizes the CIoU loss function to compute localization error, as detailed below, CIoU primarily relies on distance and shape constraints. Specifically, the CIoU loss incorporates two auxiliary components: distance loss and aspect ratio (shape) loss:

(6) $$L_{\mathrm{CIoU}}=1-\frac{B_{\mathrm{gt}}\cap B_{\mathrm{prd}}}{B_{\mathrm{gt}}\cup B_{\mathrm{prd}}}+\frac{d}{c}+a\;v$$

(7) $$d=\rho^{2}\left(B_{\mathrm{gt}},B_{\mathrm{prd}}\right)$$

(8) $$c={\left(w_{c}\right)}^{2}+{\left(h_{c}\right)}^{2}+\epsilon$$

Here, *d* represents the squared distance between the center points of the predicted box and the ground truth box, while c denotes the squared diagonal distance of the minimum bounding rectangle encompassing both boxes. The *d*/*c* term incorporates distance loss into the overall calculation. ϵ is typically set to 1 × 10^−7^ to prevent division-by-zero errors.

(9) $$a=\frac{v}{(1-\mathrm{IoU})+v}$$

(10) $$v=\frac{4{\left(\arctan\frac{w_{\mathrm{gt}}}{h_{\mathrm{gt}}}-\arctan\frac{w_{\mathrm{prd}}}{h_{\mathrm{prd}}}\right)}^{2}}{\pi^{2}}$$

**Figure 9:**
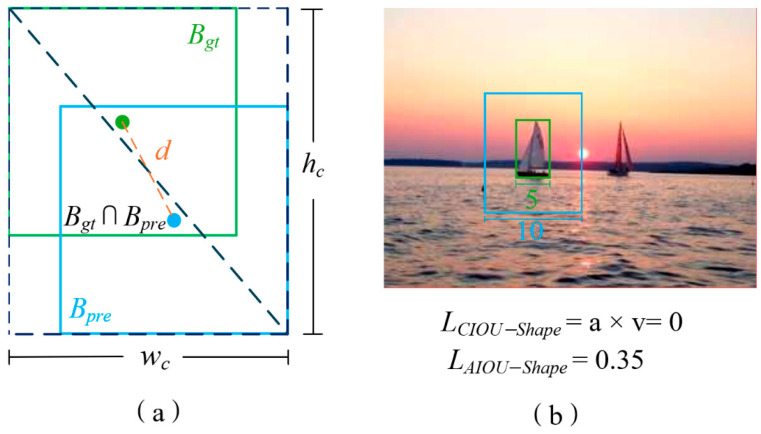
Factors influencing loss calculation. Arrows and lines indicate geometric relationships between bounding boxes. Color definitions: Green represents the ground truth bounding box (*B_gt_*) and its center point; blue represents the predicted bounding box (*B_pre_*) and its center point; dark blue dashed lines denote the minimum enclosing rectangle containing both boxes; orange denotes the center distance d. Variable definitions: *B_gt_* = ground truth bounding box, *B_pre_* = predicted bounding box, *B_gt_* ∩ *B_pre_* = intersection area of the two boxes, *d* = Euclidean distance between the centers of *B_gt_* and *B_pre_*, *wc* = width of the minimum enclosing rectangle, *hc* = height of the minimum enclosing rectangle, *L_CIOU−Shape_* = shape penalty term of CIoU loss, *L_AIOU−Shape_* = shape penalty term of the proposed AIoU loss, *α* = balance coefficient in CIoU, *v* = aspect ratio consistency term in CIoU. Subfigure (**a**) illustrates the theoretical defect of CIoU: when the predicted box and ground truth box have identical aspect ratios but different absolute sizes, the shape loss term becomes zero, providing no effective gradient guidance. Subfigure (**b**) shows a real detection scenario where AIoU computes a non-zero shape penalty by directly incorporating the absolute differences in width and height, enabling continuous optimization.

Here, v refines the shape loss by accounting for the aspect ratio difference between the predicted and ground truth boxes. However, when the aspect ratios match but sizes differ, v becomes zero, rendering shape loss ineffective (as shown in Figure 9a). In contrast, Figure 9b demonstrates that the proposed AIoU loss directly incorporates absolute width and height differences, yielding a non-zero shape penalty. AIoU overcomes this limitation by directly incorporating the absolute difference in width and height, making it more suitable for maritime detection scenarios where ship targets vary significantly in size and shape.

As shown in Equation (14), the AIoU loss function incorporates the original IoU loss, d/c distance loss, and an optimized shape loss calculation. Equations (11) and (12) compute the absolute difference between the predicted and ground-truth boxes, normalized by their maximum value to address the issue of proportionally similar boxes. Equation (13) maps the shape penalty term to the 0–1 range, thereby enhancing training speed and prediction accuracy.

The AIoU calculation process can be summarized as follows: First, the absolute differences Wdif and Hdif in width and height between the predicted and ground-truth boxes are computed. After normalization, the shape penalty term Pshape is obtained. Finally, the IoU loss, distance loss, and shape loss are combined to form the overall optimization objective. The AIoU calculation formula is shown in Equations (11)–(14):

(11) $$W_{\mathrm{dif}}=\frac{\left|{\mathrm{BoxW}}_{\mathrm{prd}}-{\mathrm{BoxW}}_{\mathrm{gt}}\right|}{\max\left({\mathrm{BoxW}}_{\mathrm{prd}},{\mathrm{BoxW}}_{\mathrm{gt}}\right)}$$

(12) $$H_{\mathrm{dif}}=\frac{\left|{\mathrm{BoxH}}_{\mathrm{prd}}-{\mathrm{BoxH}}_{\mathrm{gt}}\right|}{\max\left({\mathrm{BoxH}}_{\mathrm{prd}},{\mathrm{BoxH}}_{\mathrm{gt}}\right)}$$

(13) $$P_{\mathrm{Shape}}={\left(1-e^{-W_{\mathrm{dif}}}\right)}^{w}+{\left(1-e^{-H_{\mathrm{dif}}}\right)}^{w}$$

(14) $$L_{\mathrm{AIoU}}=1-\frac{B_{\mathrm{gt}}\cap B_{\mathrm{prd}}}{B_{\mathrm{gt}}\cup B_{\mathrm{prd}}}+\frac{d}{c}+P_{\mathrm{Shape}}$$

BoxWprd denotes the width of the predicted bounding box, BoxWgt denotes the width of the ground truth bounding box, BoxHprd denotes the height of the predicted bounding box, and BoxHgt denotes the height of the ground truth bounding box. In Equation (13), w is a relatively small constant that controls the sensitivity and weight of the shape difference terms Wdif and Hdif. Specifically, w acts as an exponent that modulates the contribution of width and height differences to the overall shape penalty PShape. Higher w values increase sensitivity to larger discrepancies, amplifying penalties for significant shape mismatches, while lower w values reduce sensitivity, diminishing penalties for minor variations. Thus, w serves as a tuning parameter to balance the impact of shape differences on the overall penalty, enabling the model to adapt to varying requirements for shape consistency.

The AIoU loss offers several advantages for maritime ship detection. By jointly optimizing overlap area, center distance, and aspect ratio, it enhances shape perception for vessels with diverse geometries, provides stable regression gradients for small-scale targets, and improves overall localization precision. These benefits are particularly critical for slender ships (e.g., sailboats) where aspect ratio plays a key role, and for small vessels that are highly sensitive to positional deviations. Consequently, AIoU strengthens model robustness in complex sea conditions involving multi-scale and variably shaped ship targets.

By jointly optimizing intersection area, center distance, and aspect ratio—a strategy specifically suited for maritime ship detection—the AIoU loss improves shape awareness for various vessel geometries, produces stable regression gradients for small-scale ships, and achieves more precise bounding box localization. This is especially critical for slender ships such as sailboats and small vessels that are sensitive to positional deviations, thereby enhancing localization robustness in complex marine environments.

### 2.6. Experimental Setup

This section details the datasets employed for model validation, the experimental configuration, and the evaluation protocols. It begins by introducing the self-built CM-Ships dataset, followed by descriptions of the public SeaShips and McShips benchmarks used to assess generalization capability. Subsequently, the implementation details—including hardware specifications, training hyperparameters, and evaluation metrics—are provided. Finally, the data augmentation strategies adopted to enhance model robustness are described.

#### 2.6.1. Self-Built CM-Ships Dataset

To bolster the model’s generalization capability across complex and dynamic maritime environments, this study constructs the CM-Ships visible-light vessel dataset through a multi-source data fusion strategy, designed for the high-fidelity simulation of authentic optical imaging conditions. This dataset integrates publicly available benchmarks, open-source imagery curated from web repositories, and field-collected maritime data. It encompasses a broad spectrum of imaging perspectives, varying spatial resolutions, heterogeneous background complexities, multi-scale target distributions, and diverse meteorological conditions to comprehensively encapsulate varied maritime scenarios.

The data sources are characterized by distinct attributes: public datasets primarily contribute imagery with nadir or standard broadside perspectives, relatively unobstructed backgrounds, and uniform target scale distributions. Web-crawled data introduces significant variations in target scale and orientation across multiple view angles and weather conditions (e.g., fog, precipitation, and clear skies). Conversely, field-collected data emphasizes intricate sea states (e.g., wave clutter and sea fog), coastal and insular backgrounds, small-scale targets, and partially occluded scenarios. The specific composition and categorical distribution characteristics of the CM-Ships dataset are summarized in Table 2.

**Figure 10:**
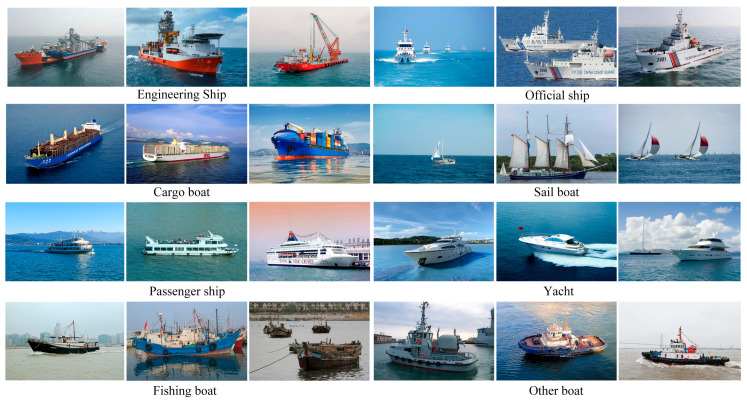
Sample images from the dataset.

The CM-Ships dataset comprises 6164 images, with native resolutions spanning from 300 × 200 to 1200 × 800 pixels. To align with the architectural input specifications of the YOLO framework, all imagery underwent a standardized preprocessing pipeline, including uniform cropping, normalization, and resizing to a consistent resolution of 640 × 640 pixels, before being archived in JPG format. The dataset categorizes maritime vessels into eight distinct categories: engineering ships, official vessels, cargo boats, passenger ships, sailboats, fishing boats, yachts, and other boats. Representative samples illustrating these categories and the diverse environmental conditions are presented in Figure 10.

To ensure dataset quality and eliminate potential training–validation leakage, near-duplicate images were removed via perceptual hashing (pHash), with a Hamming distance threshold of 5. Only one representative image was retained from each group of highly similar samples. Images acquired within a short time window and depicting the same vessel or geographic region were assigned exclusively to the training set to avoid artificially inflated model performance. Annotation consistency was strictly controlled: two experienced annotators independently labeled 10% of the dataset, and their pairwise bounding-box Intersection over Union (IoU) exceeded 0.95. Any remaining discrepancies were adjudicated by a third senior annotator. Finally, the dataset was stratified by category and randomly split into training, validation, and test sets with a ratio of 8:1:1. The number of images per category in each subset is summarized in Table 3, verifying that the original class distribution was faithfully preserved.

The distribution of categories in the CM-Ships dataset is shown in Table 3. It encompasses multi-angle, multi-scale, and long-range ship targets under common complex sea backgrounds. The images feature high resolution and clarity, with diverse target sizes, arrangements, and scene layouts. They effectively capture the typical visual characteristics of ships in visible light imagery, making the dataset suitable for multi-scale ship target detection tasks in complex marine environments.

#### 2.6.2. Public Dataset SeaShips

To further validate the generalization performance of AK-DSAM-YOLOv13, comparative experiments were conducted on the SeaShips public benchmark dataset [25]. This dataset, derived from shore-based surveillance systems, comprises 7000 images partitioned into training, validation, and testing sets with a ratio of 1750:1750:3500, respectively. The dataset encompasses six predominant vessel categories: ore carriers, bulk carriers, general cargo ships, container ships, fishing boats, and passenger ships. It systematically integrates practical challenges—including significant scale fluctuations, heterogeneous backgrounds, perspective diversity, and illumination variations—thereby effectively encapsulating the spatial and morphological distribution of vessels in authentic maritime scenarios.

**Figure 11:**
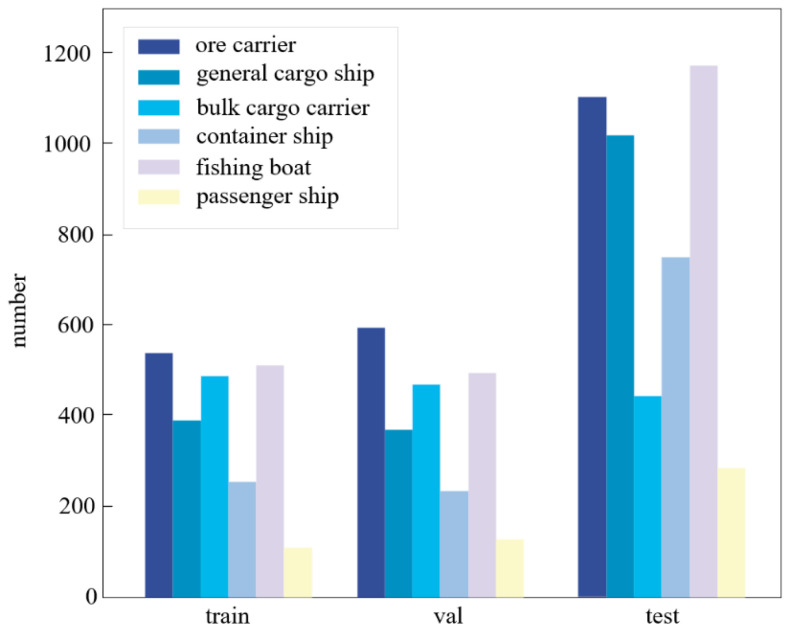
Data distribution of the SeaShips dataset.

The utilization of this benchmark facilitates a rigorous assessment of the proposed method’s robustness across divergent imaging conditions, annotation protocols, and target morphologies. This enables a comprehensive evaluation of the algorithm’s deployment potential in real-world applications. Data annotations adhere to the PASCAL VOC format and were meticulously converted to the YOLO-compliant format prior to the training phase. Samples across all taxonomies exhibit a balanced distribution across the training, validation, and test subsets, as illustrated in Figure 11.

#### 2.6.3. Public Dataset McShips

To further validate the generalization capability and universality of AK-DSAM-YOLOv13, comparative experiments were extended to the McShips public benchmark dataset [26]. Provided by Northwestern Polytechnical University, McShips augments the taxonomic scope of the SeaShips dataset [26] (from Wuhan University) by incorporating diverse military vessel categories. The dataset is characterized by heterogeneous backgrounds, substantial scale fluctuations, multifaceted perspectives, and fluctuating atmospheric and illumination conditions. It encompasses 13 distinct vessel classes with a total of 14,709 images, the categorical details of which are provided in Table 4.

McShips offers standardized partitions for training and testing, facilitating a rigorous and equitable performance evaluation of various models under a unified benchmark. The utilization of this dataset enables an assessment of the proposed method’s robustness across divergent imaging conditions, annotation protocols, and target morphologies, thereby providing a comprehensive appraisal of the algorithm’s potential for practical deployment in complex maritime scenarios.

#### 2.6.4. Experimental Environment and Evaluation Metrics

Experiments were conducted on a 64-bit Windows 10 operating system, leveraging the computational power of an NVIDIA GeForce RTX 4090 GPU. The development environment utilized the PyTorch 2.1.0 deep learning framework and Python 3.12, with CUDA 12.6 facilitating hardware acceleration. Detailed configurations for model training and hyperparameters are presented in Table 5.

To rigorously assess the detection performance of AK-DSAM-YOLOv13 across multi-scale maritime targets, the metrics of Precision, Recall, and Mean Average Precision are employed. Specifically, Precision quantifies the ratio of true positive predictions to the total number of positive predictions. Recall measures the model’s ability to correctly identify all actual positive samples. The mAP serves as the primary benchmark for holistic detection performance, representing the arithmetic mean of Average Precision (AP) across all categories. Each AP value corresponds to the area under the Precision-Recall (P-R) curve for a specific category at a designated threshold. Furthermore, the structural efficiency and degree of model lightweighting are evaluated using the total number of parameters and Giga Floating Point Operations (GFLOPs), where lower values signify reduced memory footprint and computational demand. Model inference efficiency is quantified by inference latency—defined as the time required to process a single image frame—with shorter durations indicating superior real-time execution performance.

The definitions and formulas for these metrics are as follows:

(15) $$P=\frac{\mathrm{TP}}{\mathrm{TP}+\mathrm{FP}}$$

(16) $$R=\frac{\mathrm{TP}}{\mathrm{TP}+\mathrm{FN}}$$

(17) $$AP=\left\{\begin{array}{c} \int_{0}^{1}PRdr\\ \sum_{k=1}^{n}P\left(k\right)\Delta r\left(k\right) \end{array}\right.$$

(18) $$mAP=\frac{1}{m}\sum_{i=1}^{M}AP_{i}$$

In the formula: TP represents the probability of correctly detecting a target and classifying it correctly; FP represents the probability of incorrectly detecting a non-existent target or misclassifying a target; FN represents the probability that the model fails to correctly detect an actually existing target; M represents the total number of categories in the sample. mAP50 denotes the mean average precision at an Intersection over Union (IoU) threshold of 0.5, while mAP50:95 represents the average precision across IoU thresholds ranging from 0.5 to 0.95.

#### 2.6.5. Data Augmentation Strategies

**Figure 12:**
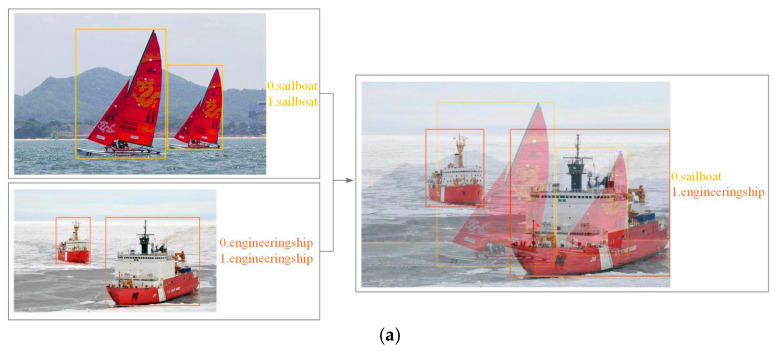

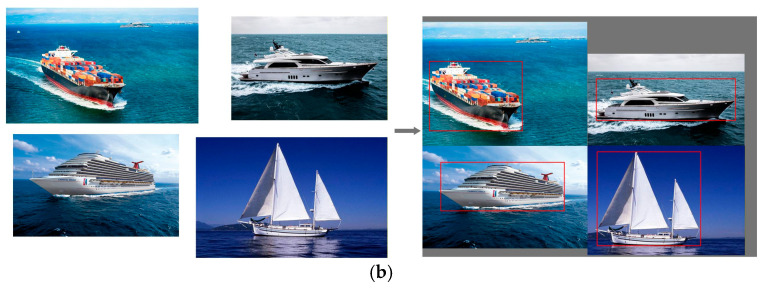
(**a**) Mixup data enhancement method. (**b**) Mosaic data enhancement method.

To bolster model generalization and mitigate overfitting, YOLOv13 integrates advanced data augmentation techniques, specifically Mixup [27] and Mosaic [28], during the training phase. Mixup expands the distribution of training samples by performing linear interpolation and pixel-wise blending of two images along with their respective annotations. This process effectively enhances the model’s adaptability to decision boundaries, as illustrated in Figure 12a. The Mosaic augmentation method involves the stochastic selection of four images, which undergo random cropping, scaling, and spatial stitching to synthesize composite training samples featuring multi-scale and multi-object scenarios. This strategy significantly increases the occurrence frequency of small-scale targets, thereby fortifying the model’s robustness against complex background interference and heterogeneous object distributions. The comprehensive workflow is depicted in Figure 12b. Notably, Mosaic augmentation operates directly at the pixel level, offering high computational efficiency and minimal hardware overhead. Consequently, this study adopts the Mosaic data augmentation strategy as a fundamental component throughout the entire training process.

## 3. Results

### 3.1. Training Process Analysis

**Figure 13:**
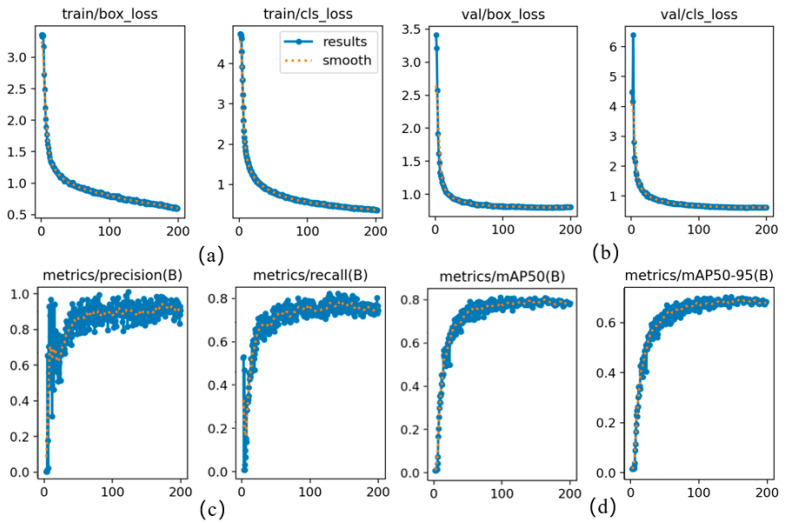
Training process curves. (**a**) Training loss curves: bounding box regression loss (train/box_loss) and classification loss (train/cls_loss); (**b**) Validation loss curves: bounding box regression loss (val/box_loss) and classification loss (val/cls_loss); (**c**) Precision and recall curves during training; (**d**) Mean average precision (mAP) curves during training, including mAP50 and mAP50:95. The x-axis represents the number of training epochs (0–200). Blue solid lines denote raw experimental results, and orange dashed lines denote smoothed results.

Figure 13 presents the comprehensive training dynamics of the AK-DSAM-YOLOv13 model, including loss function curves (training and validation), Precision-Recall (P-R) curves, and Mean Average Precision (mAP) trajectories. Analysis of the loss profiles reveals that the model exhibits exceptional optimization efficiency and superior convergence stability. Specifically, during the initial training phase (first 50 epochs), both training and validation losses decrease precipitously, benefiting from a cosine annealing learning rate schedule and gradient clipping. In the mid-iteration phase (50–100 epochs), the rate of decline decelerates as the model enters a fine-tuning stage, characterized by the acquisition of fine-grained categorical features. During the late training phase (100–200 epochs), the model converges to a stable equilibrium around the 200th epoch, with the generalization gap—the discrepancy between validation and training losses—stabilizing within a minimal margin. Notably, the validation loss curve exhibits high fidelity to the training curve without significant oscillations or divergence, suggesting that the model effectively mitigates overfitting risks and maintains robust generalization capabilities. The evaluation metric curves demonstrate simultaneous improvements in Precision and Recall, which ultimately saturate at high-performance levels of approximately 0.93 and 0.84, respectively. This validates the model’s ability to achieve an optimal trade-off between detection accuracy and recall comprehensiveness. Furthermore, mAP50 and mAP50:95 converge to 0.809 and 0.702, respectively. This high-fidelity performance under stringent IoU thresholds further validates the efficacy of the Accuracy-Intersection-over-Union (AIoU) loss module in enhancing bounding box regression precision. It underscores the model’s capacity to meet the rigorous requirements for the precise localization of small-scale, high-clutter ship targets within complex maritime environments.

The detection precision for individual vessel categories is summarized in Table 6. Statistical analysis of the detection performance across various ship targets further substantiates the model’s exceptional capabilities. The AK-DSAM-YOLOv13 architecture demonstrates superior detection performance across multiple vessel classes, achieving an overall mean Average Precision (mAP) of 87.7%. Furthermore, the average inference latency per image is approximately 3.2 milliseconds, effectively satisfying the requirements for real-time maritime surveillance tasks. Regarding category-specific performance, engineering vessels and official vessels (government ships) achieve superior detection results. This is primarily attributed to their larger pixel footprint and distinct morphological and structural attributes, which provide prominent feature saliency. Consequently, the model can readily extract stable and discriminative representations for these categories.

In contrast, the detection accuracy for fishing boats and other boats is slightly lower than that of other categories. This discrepancy arises from the high intraclass variance and irregular geometric profiles of fishing boats; furthermore, small-scale fishing boats exhibit limited feature coverage, increasing their susceptibility to misclassification. Similarly, the “other boats” category encompasses a highly heterogeneous set of targets lacking unified structural features, which complicates the model’s ability to converge on stable discriminative rules. Notably, sailboat detection achieves an exceptional precision of 0.984, despite their predominantly small-scale representation within the dataset. This result underscores the model’s robust performance in small-object detection tasks and validates the efficacy of the proposed architectural enhancements.

**Figure 14:**
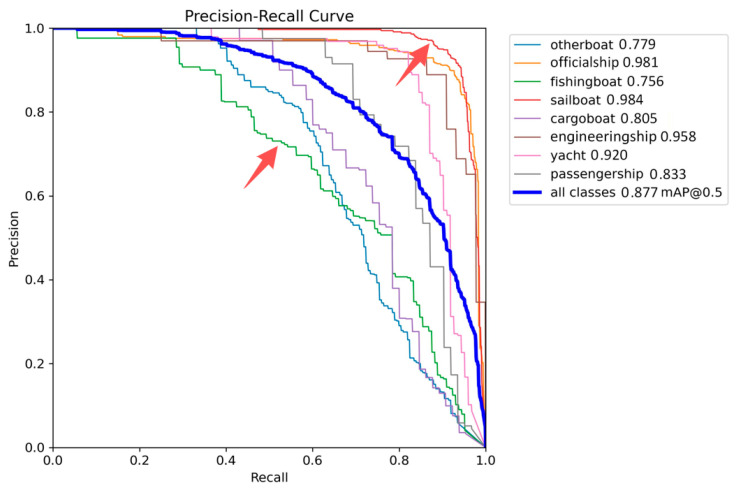
Precision-Recall (PR) curves of the proposed AK-DSAM-YOLOv13 model on the CM-Ships dataset. The x-axis represents recall, and the y-axis represents precision. Red arrows highlight two representative ship categories: the upper arrow points to the sailboat curve, which achieves the highest detection accuracy (mAP50 = 0.984); the lower arrow points to the fishingboat curve, which has relatively lower detection performance (mAP50 = 0.756). The overall mAP50 across all classes is 0.877.

**Figure 15:**
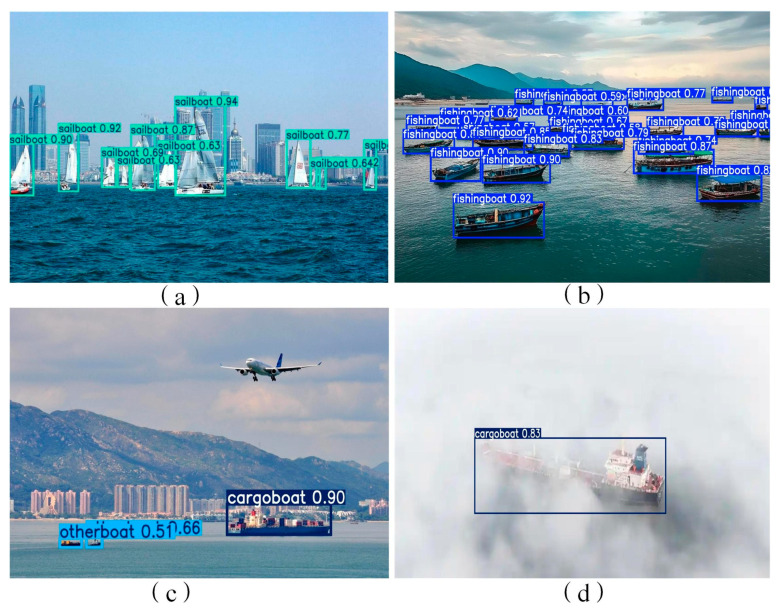
Actual detection results of the network. (**a**) coastal clutter background; (**b**) dense overlapping ships; (**c**) mixed-scale and multi-class vessels; (**d**) low-visibility sea fog environment.

The Precision-Recall (PR) curves of the enhanced model are depicted in Figure 14. The graphical representation indicates that the PR trajectories for engineering vessels and sailboats are most proximal to the top-right coordinate of the plot, signifying the highest detection accuracy for these two specific categories. Furthermore, the curves for the remaining six vessel types maintain elevated positions across the recall spectrum, demonstrating a consistently superior detection performance across all taxonomies. The proposed AK-DSAM-YOLOv13 network exhibits strong performance in complex maritime ship detection, and its comprehensive capabilities are fully validated by the qualitative results illustrated in Figure 15. Specifically, as shown in Figure 15a, the model effectively suppresses strong coastal interference from shoreline structures while accurately identifying all sailboat targets, ensuring reliable detection in high-clutter marine backgrounds. In the dense berthing scenario shown in Figure 15b, the network robustly handles severe mutual occlusion among closely docked vessels, generating clear and well-separated bounding boxes for each target while maintaining stable localization and classification accuracy. For the mixed-scale scenario in Figure 15c, which includes large vessels, small targets, and multiple ship classes, the model successfully detects all objects—from large cargo ships to tiny vessels occupying only a few pixels—while precisely distinguishing different ship categories and preserving reliable localization even for targets with extremely limited edge information. Furthermore, under degraded visibility caused by dense sea fog in Figure 15d, the model still detects targets reliably despite significant visual degradation, demonstrating its strong environmental adaptability and robustness. Collectively, these qualitative results confirm that the proposed network maintains stable and accurate detection performance against the typical challenges of complex marine environments, including background clutter, target occlusion, multi-class coexistence, small-scale targets, and severe weather conditions.

### 3.2. Comparative Experiments

All competing models were trained and tested under identical hardware configurations and hyperparameter settings, as detailed in Section 2.6.4. Evaluations were performed on three datasets: the self-built CM-Ships dataset (Section 3.2.1), the public SeaShips benchmark (Section 3.2.2), and the McShips dataset (Section 3.2.3).

#### 3.2.1. Comparison Based on the CM-Ships Dataset

To evaluate the overall performance of the proposed AK-DSAM-YOLOv13 under diverse marine conditions, comparative experiments were carried out on the CM-Ships dataset with several mainstream single-stage detectors. Given that this dataset is constructed for real-time maritime monitoring and edge deployment, we focus the comparison on lightweight YOLO-series models (YOLOv8n–YOLOv13n), which provide a practical trade-off between accuracy and speed. The quantitative results are presented in Table 7. Comparative analysis demonstrates that AK-DSAM-YOLOv13 achieves superior performance across multiple key evaluation metrics. Specifically, its Precision (P) reaches 88.4%, and the mean Average Precision at a 0.5 IoU threshold (mAP50) is 87.7%, representing improvements of 1.9 and 1.4 percentage points over the baseline YOLOv13n model, respectively. Notably, the Recall (R) stands at 80.7%, surpassing all compared models. These results validate that the AKC3k2 module, by leveraging Alterable Kernel Convolution (AKConv), achieves adaptive receptive field modulation. This mechanism effectively enhances the extraction of local features from small-scale objects, thereby significantly mitigating false-negative rates. Regarding computational efficiency, AK-DSAM-YOLOv13 comprises 3.84 million parameters and 6.8 GFLOPs, with an average inference latency of 3.2 ms. Compared to YOLOv8n (3.11M parameters, 10.6 GFLOPs, 3.4 ms) and YOLOv9n (3.70M parameters, 11.8 GFLOPs, 3.8 ms), the proposed approach achieves higher detection accuracy while maintaining a highly competitive computational overhead. Although the integration of dynamic upsampling and attention mechanisms within the DyDSAM module incurs a marginal computational cost, the 3.2 ms single-frame processing speed remains well below the temporal threshold required for real-time maritime monitoring systems. This underscores an optimal trade-off between detection precision and execution speed. In summary, AK-DSAM-YOLOv13 achieves higher precision, recall, and mAP compared to all lightweight YOLO baselines on the CM-Ships dataset.

**Figure 16:**
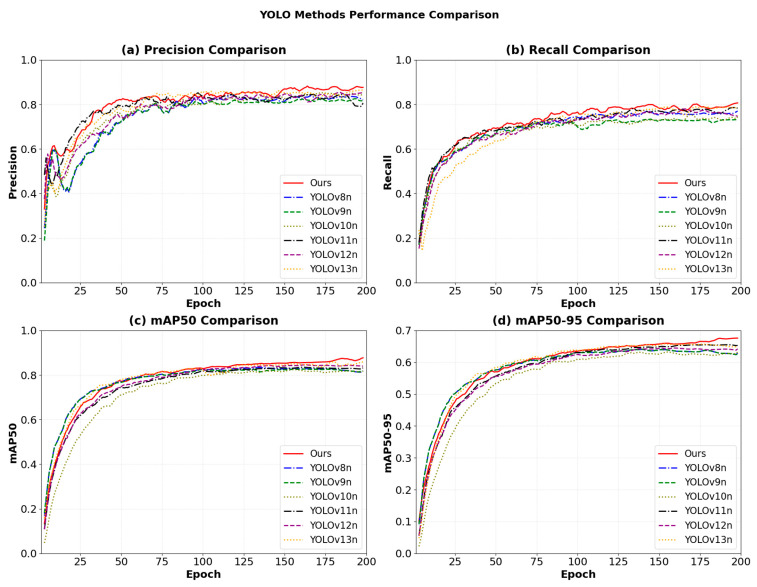
Detection results of comparative experiments.

Figure 16 illustrates the evolution trajectories of various performance metrics across different models during the training phase. As demonstrated in Figure 16a,b, AK-DSAM-YOLOv13 achieves a rapid ascent in both Precision and Recall during the initial training stage (the first 50 epochs). This “cold-start” advantage is primarily attributed to the optimized loss formulation, which models bounding boxes as two-dimensional Gaussian distributions to provide a smoother gradient flow, thereby effectively mitigating gradient oscillations during the early-stage training of small-scale targets. In contrast, YOLOv8 encounters a performance plateau after 100 epochs, whereas the proposed method maintains continuous optimization potential throughout the entire training cycle.

As depicted in Figure 16c, the proposed method exhibits the most accelerated growth in mAP50, significantly surpassing all baseline models after approximately 25 epochs and maintaining a consistent lead at approximately 0.85. While YOLOv8 shows a minor early peak, its performance improvement subsequently stagnates. YOLOv11 and YOLOv13 demonstrate moderate performance, while YOLOv9 and YOLOv10 relatively lag behind. Regarding the mAP50:95 metric (Figure 16d), which more accurately reflects localization quality, AK-DSAM-YOLOv13 establishes a substantial lead after 80 epochs and preserves this superiority until training completion. This suggests that the geometric correction facilitated by DySample within the DyDSAM structure contributes to improved regression accuracy for vessel boundaries and may help mitigate the localization drift issues inherent in traditional interpolation methods.

**Figure 17:**
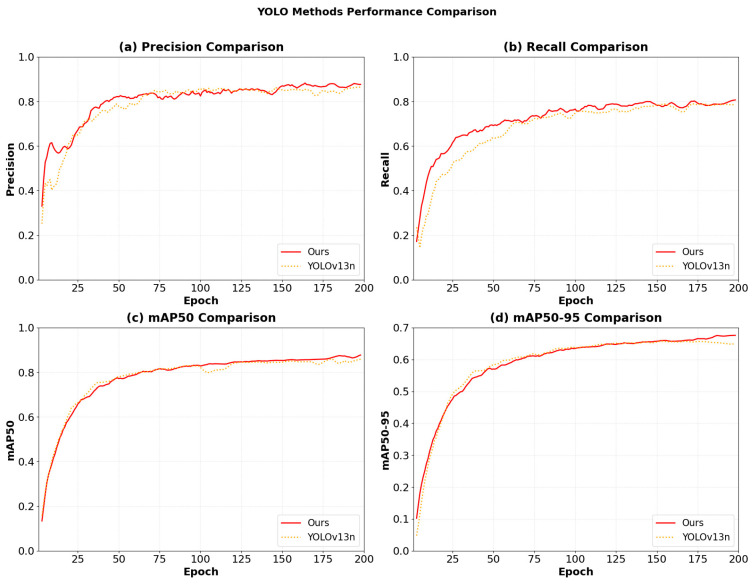
Comparison curves between the proposed method and the original YOLOv13n.

Figure 17 further provides a direct comparison of the model performance before and after ablation. The substantial margin between the curves visually quantifies the cumulative gains derived from the AKC3k2, DyDSAM, and AIoU-Loss modules. This confirms that the proposed integrated strategy forms a robust feature-enhancement closed-loop, significantly bolstering the model’s efficacy for ship detection in complex environments.

In summary, AK-DSAM-YOLOv13 achieves optimal values across four core metrics: accuracy, recall, mAP50, and mAP50:95. Although its model parameters and computational requirements are slightly higher than some models, experiments demonstrate that this minor computational overhead yields significant improvements in accuracy and robustness while still meeting real-time detection requirements.

#### 3.2.2. Experimental Comparison Using the SeaShips Dataset

To evaluate the generalization capability of the proposed model on public benchmarks, comprehensive comparative experiments were conducted on the SeaShips dataset. In contrast to the evaluation on the CM-Ships dataset, the comparison is extended to include two-stage detectors (Faster R-CNN [29], Libra R-CNN [30]), a representative single-stage detector (SSD [31]), an anchor-free detector (FCOS [32]), and Transformer-based detectors (Deformable DETR [33], DINO [34]), in addition to the YOLO-series models. This comprehensive comparison enables a thorough evaluation of the proposed method against a wide range of state-of-the-art detection frameworks. The quantitative results of all compared models are summarized in Table 8.

As summarized in Table 8, the proposed AK-DSAM-YOLOv13 achieves strong performance across multiple core evaluation metrics. Its Precision reaches 94.6%, and its Recall reaches 91.4%, both ranking highest among all compared models, indicating effective mitigation of false negatives. Regarding detection accuracy, mAP50 and mAP50:95 are 95.1% and 76.3%, respectively—the highest values among all evaluated models. The model maintains its lead under the more stringent mAP50:95 metric, demonstrating superior localization robustness. These results suggest that even on a high-quality dataset such as SeaShips, the AIoU loss effectively enhances bounding box regression accuracy. By jointly optimizing overlap area, center distance, and aspect ratio, the module improves localization precision, which is especially beneficial for small-scale targets whose bounding boxes tend to be more sensitive to positional deviations.

In terms of model efficiency, AK-DSAM-YOLOv13 exhibits excellent lightweight characteristics: its parameter count (3.84 M) is substantially lower than that of two-stage detectors and Transformer architectures, and also ranks among the lowest in the lightweight YOLO series. Its computational complexity (6.8 GFLOPs) lies between YOLOv11n (6.6 GFLOPs) and YOLOv12n (7.2 GFLOPs), indicating that overhead remains well controlled after integrating the AKC3k2 and DyDSAM modules. The single-frame inference time (3.2 ms) is slightly higher than that of YOLOv10n (2.3 ms) and YOLOv13n (2.4 ms) but is substantially lower than that of computationally intensive models (e.g., Faster R-CNN, DINO), fully meeting the real-time requirements of maritime surveillance systems.

**Figure 18:**
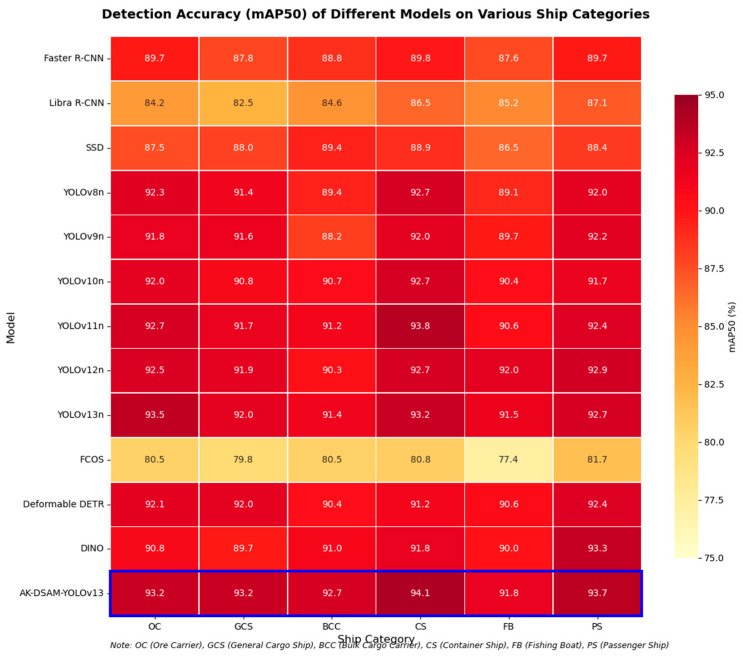
Heatmap comparison of detection accuracy (mAP50) across ship categories. The color bar on the right indicates the mAP50 value (%), with darker red representing higher detection accuracy. The blue border highlights the results of the proposed AK-DSAM-YOLOv13 model. Abbreviations for ship categories are defined in the note below the figure.

To analyze the model’s adaptability to different vessel types, Figure 18 presents a heatmap comparing fine-grained classification detection accuracy (mAP50).

Heatmap analysis reveals that AK-DSAM-YOLOv13 (bottom row) exhibits the most intense color representation across the majority of vessel categories, reflecting its superior overall generalization performance. Specifically, the proposed model achieves the highest precision among all evaluated architectures for container ships (CS, 94.1%) and passenger ships (PS, 93.7%). It also ranks first for general cargo ships (GCS, 93.2%) and bulk carriers (BCC, 92.7%). Regarding ore carriers (OC), its accuracy (93.2%) is marginally lower than the top-performing YOLOv13n (93.5%) but remains within the leading tier. Notably, in the challenging fishing boat (FB) category, its precision (91.8%) closely approximates that of the best-performing YOLOv12n (92.0%) and significantly outperforms most other baseline models. This validates the capacity of the AKC3k2 module to effectively enhance the feature representation of small-scale and low-contrast targets.

A comparative analysis shows that two-stage detectors (e.g., Faster R-CNN) exhibit lighter color intensities across all categories, while YOLO series models show stronger performance. AK-DSAM-YOLOv13 achieves the most balanced and consistently high-performance color profile across all vessel categories.

#### 3.2.3. Experiments Based on the McShips Dataset

To further validate the generalization capability of the proposed model in complex scenarios with high intra-class diversity, extended comparative experiments were carried out on the McShips dataset. Following the same evaluation protocol adopted for the SeaShips dataset, we compare with a wide range of state-of-the-art-detectors, including two-stage detectors (Faster R-CNN, Libra R-CNN), a representative single-stage detector (SSD), an anchor-free detector (FCOS), and Transformer-based detectors (Deformable DETR, DINO), in addition to the YOLO-series models. The quantitative experimental results are presented in Table 9.

**Figure 19:**
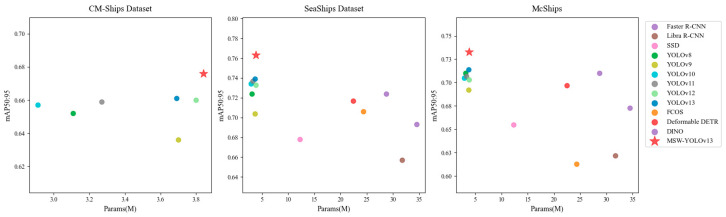
Comparison of model performance (mAP50:95 vs. number of parameters) across the CM-Ships, SeaShips, and McShips datasets.

To further illustrate the trade-off between detection accuracy and model complexity, we focus on two key metrics: mAP50:95 (comprehensive localization precision) and the number of parameters (model size). These metrics are jointly visualized in Figure 19, where each model is represented as a point in the accuracy–complexity space across the CM-Ships, SeaShips, and McShips datasets. This two-dimensional perspective enables an intuitive evaluation of the accuracy–efficiency balance achieved by each model.

As illustrated, AK-DSAM-YOLOv13 achieves higher mAP50:95 with fewer parameters than Transformer-based models and most large-scale CNN frameworks, while also outperforming lightweight approaches that sacrifice accuracy for reduced complexity.

In summary, AK-DSAM-YOLOv13 achieves higher precision, recall, and mAP than all compared methods on the SeaShips and McShips datasets, including two-stage detectors, anchor-free detectors, and Transformer-based architectures.

### 3.3. Ablation Studies

To evaluate the individual contribution of each proposed enhancement to the overall model performance, systematic ablation experiments were conducted on the CM-Ships dataset. All ablation experiments were performed under a fixed random seed to ensure single-variable control. The baseline YOLOv13n results in the ablation experiments are consistent with those in the comparative experiments within experimental variation, with no statistical difference. Taking the original YOLOv13n as the baseline, we sequentially integrated Alterable Kernel Convolution (AKConv), DySample dynamic upsampling, the Dual-Stream Attention Module (DSAM), and the Accuracy-Intersection-over-Union (AIoU) loss function. The impact on detection precision and computational efficiency was quantitatively analyzed, as summarized in Table 10.

The individual implementation of these modules yielded the following results:

AKConv Integration: The integration of AKConv alone increased the mAP50 from 86.5% to 86.7%, while simultaneously reducing the GFLOPs from 6.8G to 6.5G. This proves that the module successfully augments feature extraction capabilities while effectively mitigating computational complexity.

DySample Integration: Applying DySample dynamic upsampling independently improved the mAP50:95 to 66.7% with only a marginal increase in inference latency. This suggests that its content-aware resampling mechanism enhances detail retention, thereby providing a superior spatial foundation for subsequent feature fusion.

DSAM Integration: The incorporation of the DSAM module further bolstered the mAP50 to 86.7%, validating the efficacy of the dual-stream attention mechanism in suppressing background interference and enhancing target saliency.

AIoU Loss Implementation: Adopting the AIoU loss function resulted in an mAP50 of 86.4% without affecting the model’s parameter count or computational cost. The standalone AIoU loss yields only a modest gain, as it primarily refines bounding box regression rather than enhancing feature extraction. When combined with AKC3k2 and DyDSAM, its full potential is realized, contributing to the notable localization improvements in the full model.

As the modules were progressively integrated, the model performance exhibited a consistent monotonic improvement. When AKConv, DySample, DSAM, and AIoU were fully integrated, the comprehensive performance reached its zenith: mAP50 increased to 88.4% (a 1.9 percentage point improvement over the baseline), and mAP50:95 rose to 67.6%. The final architecture maintains a lightweight footprint with 3.84 million parameters, 6.8 GFLOPs, and a single-frame inference latency of 3.2 ms. These results indicate that the integrated design yields cumulative improvements that exceed the sum of individual gains, suggesting effective complementarity among the proposed modules. In summary, AK-DSAM-YOLOv13 achieves substantial gains in detection precision and robustness within complex maritime scenarios with minimal additional inference overhead, through the combined integration of AKConv-based feature enhancement, DySample upsampling optimization, DSAM attention fusion, and AIoU loss constraints.

To further evaluate the advantage of AIoU for ship detection, we compared it with five commonly used IoU-based losses: CIoU [35], DIoU [36], GIoU [37], EIoU [38], and WIoU [39]. All models shared the same architecture (YOLOv13n + AKConv + DySample + DSAM) and training settings; only the loss function was varied. As shown in Table 11, AIoU achieves the highest mAP50:95 (67.6%), outperforming CIoU (67.2%) by 0.4 percentage points. It also achieves the best mAP50 (87.7%) and recall (80.7%). The other losses yield intermediate results, all lower than AIoU. These results suggest that AIoU provides more stable gradient guidance for bounding box regression, particularly for small and occluded ship targets. The small differences in inference time among all loss functions (3.0–3.2 ms) are within the normal measurement fluctuation range of the GPU. All results satisfy the real-time requirement (≤10 ms per image). Since the loss function is not executed during inference, the accuracy improvement of AIoU does not introduce any additional computational overhead at test time.

To further evaluate the effectiveness of DySample relative to alternative upsampling methods under a unified architecture, we conducted an ablation study while fixing the backbone to YOLOv13n + AKConv + DSAM + AIoU (i.e., the full model without DySample). The upsampling operator was then replaced with bilinear interpolation [40], bicubic interpolation [41], CARAFE [18], and DySample [21], respectively, with all other training settings kept identical. The quantitative results are presented in Table 12.

Among the evaluated upsampling strategies, DySample achieves the highest mAP50 of 87.7% and mAP50:95 of 67.6%, representing a 0.4 percentage point improvement over the baseline nearest-neighbor interpolation. Although CARAFE yields a slightly higher precision of 88.5% than DySample (88.4%), it lags behind DySample in both mAP50 (87.6% vs. 87.7%) and mAP50:95 (67.4% vs. 67.6%), while introducing substantially higher latency (3.5 ms vs. 3.2 ms) and computational cost (7.2 GFLOPs vs. 6.8 GFLOPs). In contrast, bilinear and bicubic interpolation introduce negligible changes to model parameters and computational load. However, their detection accuracy is slightly lower than DySample (e.g., mAP50:95 of 67.3% vs. 67.6%), suggesting that the inherent smoothing nature of traditional interpolation may degrade the preservation of fine-grained vessel boundaries. This degradation is particularly critical in maritime scenarios, where distinguishing closely berthed ships and localizing small/distant targets relies heavily on sharp edge information. By jointly considering detection accuracy, computational efficiency, and inference latency, DySample offers the most favorable performance trade-off, making it highly suitable for real-time maritime surveillance applications.

To further quantify the effectiveness of AKC3k2 on small-scale ship targets, we conducted a dedicated single-module replacement experiment, as summarized in Table 13.

Despite incurring only a marginal 5.1% increase in parameters and a 3.7% increase in computational cost, AKC3k2 improves mAP50 by 4.4 percentage points and recall by 5.3 percentage points for small-scale ship detection. Inference time increases by only 0.4 ms, still satisfying real-time maritime detection requirements. This further confirms that AKC3k2’s dynamic receptive field is particularly beneficial for capturing small-vessel features.

### 3.4. Visual Analysis

#### 3.4.1. Visualization of Detection Results

**Figure 20:**
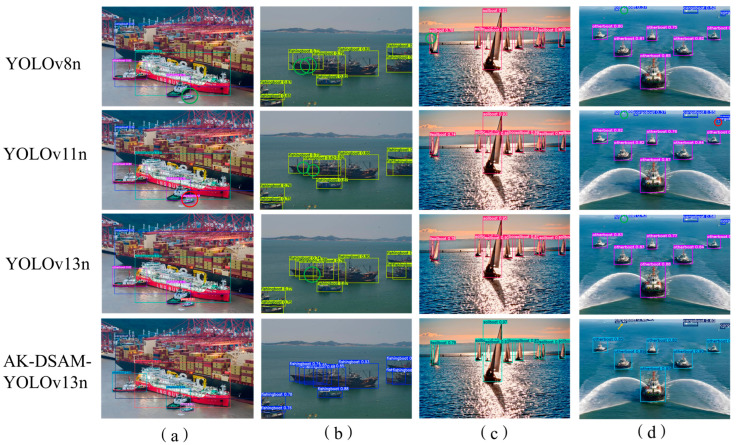
Visual comparison of generalization ability on the CM-Ships dataset. Red circles indicate false positives (FP), green circles indicate false negatives (FN), and yellow arrows mark targets that are only successfully detected by the proposed AK-DSAM-YOLOv13. (**a**) Dense port scene; (**b**) Occluded fishing boat scene; (**c**) Strong backlight scene; (**d**) Foggy offshore scene.

Figure 20 compares AK-DSAM-YOLOv13 with YOLOv8n, YOLOv11n, and YOLOv13n on four challenging scenes from the CM-Ships dataset. Red circles mark false positives, green circles mark false negatives, and yellow arrows denote targets detected only by our model. In the dense port scene (a), YOLOv8n misses a small speedboat and YOLOv11n misclassifies it, while YOLOv13n yields low confidences (<0.8) for small targets. Our model correctly detects all vessels with high confidence, benefiting from AKConv’s adaptive receptive field and DSAM’s background suppression. In the occluded fishing boat scene (b), YOLOv8n misses three boats and produces repeated detections, YOLOv11n misses two, and YOLOv13n misses one with confidences below 0.6. Our model detects all boats without misses or duplicates (confidences > 0.65, up to 0.93), thanks to AK-Bottleneck fine-grained feature extraction and enhanced multi-scale fusion. Under strong backlight (c), YOLOv8n misses the leftmost sailboat, while the other three methods detect it, but our model achieves the highest confidences (e.g., 0.97 for dense sailboats on the right), enabled by DSAM glare suppression and dynamic upsampling. In the foggy offshore scene (d), YOLOv8n misses the distant small vessel, YOLOv11n produces two false positives, and YOLOv13n also misses the distant target. Only our model detects the far-distance vessel (yellow arrow) and attains 0.95 confidence for the near-distance ship, demonstrating effective sea-clutter filtering via DSAM and enhanced shallow features via multi-scale fusion. Overall, our model consistently achieves higher recall, better localization, and stronger background suppression across all scenarios.

**Figure 21:**
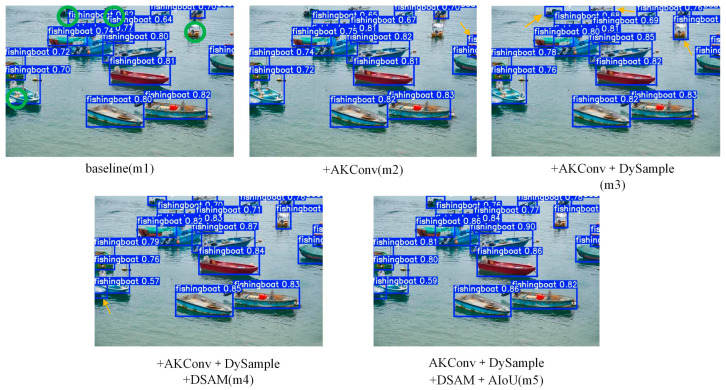
Actual detection results of ablation experiments. Green circles denote false negatives (FN, missed vessel targets), and yellow arrows mark newly recovered targets after adding each module. (**m1**) Baseline YOLOv13n; (**m2**) Baseline + AKConv; (**m3**) m2 + DySample; (**m4**) m3 + DSAM; (**m5**) Full AK-DSAM-YOLOv13 model.

Figure 21 visualizes the progressive performance improvement of each proposed module via ablation study. Green circles denote false negatives (FN, missed vessel targets), and yellow arrows mark the newly recovered targets enabled by the added module. The baseline model (m1) misses five vessels in the dense and occluded scenario. Adding AKConv (m2) successfully recovers one missed target (yellow arrow), demonstrating its capability to capture fine-grained features of small-scale vessels. Further integrating DySample (m3) recovers three additional missed vessels (yellow arrows), with significantly tighter bounding boxes that better align with the actual contours of ships, verifying its effectiveness in improving localization accuracy. With the addition of the DSAM module (m4), the last remaining missed target is detected (yellow arrow), achieving full recovery of all five previously missed vessels; the bounding boxes are further refined to match vessel boundaries more precisely, benefiting from DSAM’s background clutter suppression and feature enhancement. Finally, the full model (m5) achieves complete and accurate detection of all vessels with precise bounding box positioning, eliminating all false negatives. The progressive reduction of missed targets (green circles) and the sequential recovery of vessels (yellow arrows) directly and intuitively validate the independent contribution of each proposed module.

**Figure 22:**
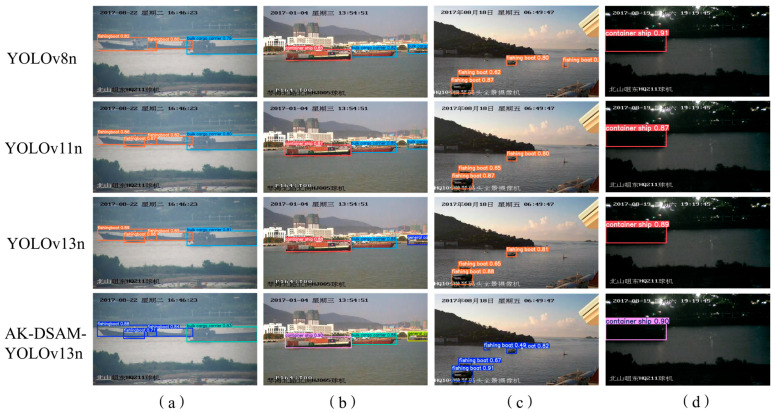
Visual comparison of generalization ability on the SeaShips dataset. (**a**) Cluttered port scenario; (**b**) Target occlusion scenario; (**c**) Small-scale vessel scenario; (**d**) Low-illumination night scenario. The proposed AK-DSAM-YOLOv13 achieves better bounding box alignment, higher occlusion detection accuracy, and full small-target coverage compared to baseline YOLO models. The Chinese text in the images originates from the original SeaShips dataset and does not interfere with the detection comparison or the scientific conclusions.

To further verify the cross-dataset generalization capability of the proposed AK-DSAM-YOLOv13 on public maritime detection benchmarks, Figure 22 provides a qualitative visual comparison between the proposed model and three state-of-the-art lightweight YOLO baselines (YOLOv8n, YOLOv11n, and YOLOv13n) on the SeaShips dataset. Qualitative analysis across diverse challenging scenarios is detailed as follows:

Cluttered port scenario (Figure 22a): All baseline models only achieve basic vessel localization with loose bounding boxes, while the proposed AK-DSAM-YOLOv13 exhibits significantly better spatial alignment with vessel contours and higher bounding box localization precision.

Target occlusion scenario (Figure 22b): YOLOv11n completely misses the occluded vessel targets, while YOLOv8n and YOLOv13n produce categorical misclassifications for partially obscured ships. In contrast, the proposed AK-DSAM-YOLOv13 accurately predicts both the precise bounding box coordinates and correct class labels for all occluded targets.

Small-scale vessel scenario (Figure 22c): YOLOv8n and YOLOv13n only detect three nearshore small targets, YOLOv11n identifies only two, and all baselines miss distant tiny vessels. By contrast, the proposed AK-DSAM-YOLOv13 achieves full and accurate detection of all small-scale vessels, including distant tiny targets ignored by the baselines.

Low-illumination night scenario (Figure 22d): All compared models complete valid detection and classification of vessel targets. Although the proposed AK-DSAM-YOLOv13 has slightly lower confidence scores in individual cases compared with YOLOv8n, it maintains a prominent lightweight advantage: its parameter count and computational complexity are significantly optimized while achieving comparable detection performance in harsh low-light conditions.

Overall, the qualitative comparison results demonstrate that the proposed AK-DSAM-YOLOv13 has stronger environmental adaptability and structural robustness across various challenging maritime scenarios, and achieves better comprehensive detection performance than mainstream lightweight YOLO baselines on the public SeaShips dataset. This further validates the excellent cross-dataset generalization capability of the proposed model.

**Figure 23:**
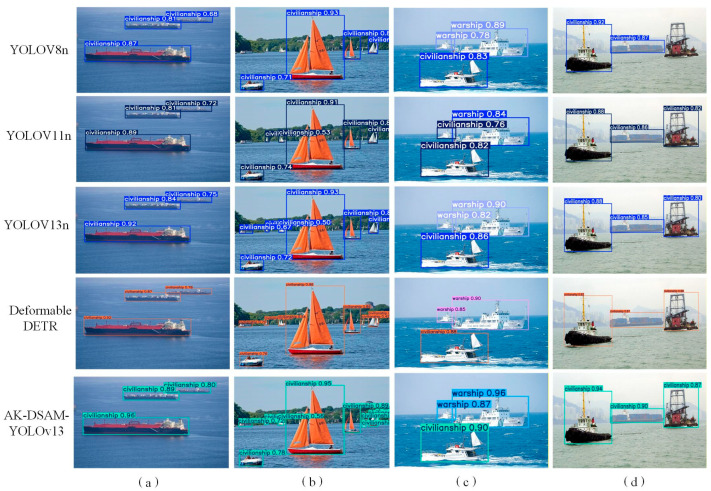
Visual comparison of generalization ability on the McShips dataset. (**a**) Complex offshore scenario; (**b**) Dense multi-target scenario; (**c**) Multi-scale vessel scenario; (**d**) Complex port background scenario. The proposed model achieves higher prediction confidence (up to 0.96), precise localization, and full detection across all scales.

To further validate the cross-dataset generalization capability and universality of the proposed AK-DSAM-YOLOv13 on public maritime detection benchmarks, Figure 23 provides a qualitative visual comparison of detection performance between the proposed model and four state-of-the-art detectors (YOLOv8n, YOLOv11n, YOLOv13n, and the Transformer-based Deformable DETR) on the McShips dataset. Qualitative analysis across diverse challenging scenarios is detailed as follows:

Complex offshore scenario (Figure 23a): All baseline models only achieve basic vessel localization with loose bounding boxes and limited prediction confidence. In contrast, the proposed AK-DSAM-YOLOv13 achieves significantly higher prediction confidence (up to 0.96) and more precise spatial localization, with bounding boxes tightly aligned to the actual contours of ship targets.

Dense multi-target scenario (Figure 23b): YOLOv11n and Deformable DETR suffer from severe target omissions and categorical misclassifications for small sailboats, while YOLOv8n and YOLOv13n produce markedly low confidence scores for small-scale targets. By comparison, the proposed AK-DSAM-YOLOv13 accurately predicts the precise spatial coordinates and correct categorical identities of all targets in the dense scene, with stable and reliable confidence scores even for tiny vessels.

Multi-scale vessel scenario (Figure 23c): The YOLOv8n, YOLOv11n, and YOLOv13n baselines fail to detect partial small-scale vessels, and Deformable DETR exhibits obvious categorical misclassifications for warship targets. The proposed AK-DSAM-YOLOv13 achieves full and accurate detection of ship targets across all scales, with high classification fidelity for both large vessels and tiny distant targets.

Complex port background scenario (Figure 23d): The proposed AK-DSAM-YOLOv13 consistently delivers higher detection confidence than all baseline models. More importantly, it maintains a decisive lightweight advantage over the compared models: it achieves superior detection performance with far fewer parameters and lower computational complexity than both YOLO-series baselines and the larger Deformable DETR, achieving an optimal trade-off between real-time inference efficiency and detection accuracy.

Overall, the qualitative comparison results demonstrate that the proposed AK-DSAM-YOLOv13 exhibits superior environmental adaptability and structural robustness across various challenging maritime scenarios, outperforming the selected state-of-the-art baselines in comprehensive detection performance. These visualization results are fully consistent with the quantitative experimental results on the McShips dataset, further verifying the excellent cross-dataset generalization capability of the proposed model.

#### 3.4.2. Feature Heatmap Visualization

**Figure 24:**
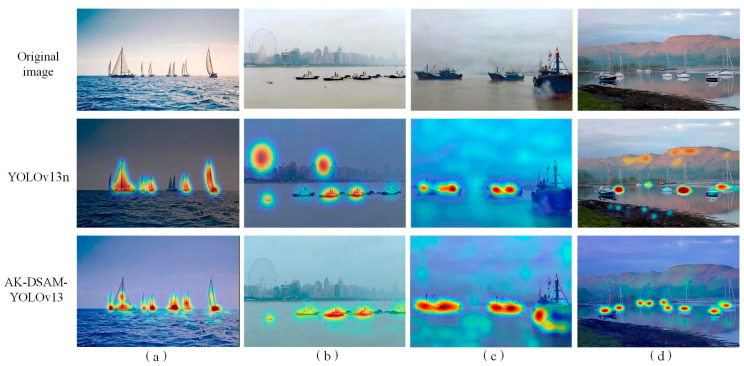
Grad-CAM++ heatmap visualization comparing feature attention between baseline YOLOv13n and the proposed AK-DSAM-YOLOv13. Red/yellow regions indicate high activation (strong focus on ship targets), while blue indicates low activation (suppressed background). (**a**) Small-scale sailboat scenario; (**b**) Urban background interference; (**c**) Low-contrast sea fog; (**d**) Dense coastal vessel scenario. The proposed model achieves concentrated, precise activation aligned with vessel contours.

To validate the augmented feature capture capability of the proposed model, Grad-CAM++ [42] was employed to generate heatmaps, visualizing the model’s primary regions of interest (RoIs) within input imagery. Figure 24 presents a comparative visualization of heatmaps generated by the baseline and the improved models across multi-scale maritime scenarios, where the pixel-wise activation magnitude reflects the model’s attentional weight assigned to corresponding regions.

The visualization results demonstrate that the proposed model exhibits significantly superior feature saliency focusing capability over the baseline:

Small-scale sailboat scenario (Figure 24a): The baseline model shows relatively dispersed feature responses, with redundant activations on non-target sea surface backgrounds. Meanwhile, the activation intensity for distant small sailboats is severely attenuated with limited spatial coverage, leading to insufficient feature representation and a high risk of missed detection. In contrast, the proposed model precisely concentrates feature responses on all small sailboats. It not only substantially amplifies the activation intensity of tiny targets, but also accurately covers the complete structural contour of each vessel, achieving efficient feature acquisition for small-scale targets, which benefits from the dynamic receptive field optimization of AKConv.

Urban background interference scenario (Figure 24b): When dealing with scenes where vessels are juxtaposed with urban shoreline buildings, the baseline model suffers from severe background distraction. It produces high-intensity false-positive activations in non-target building regions, leading to the dilution of target-specific feature representation. The proposed model shows negligible weak responses to complex backgrounds, and its activation intensity and coverage completeness for vessel targets are significantly better than the baseline. It accurately discriminates ship targets from interfering backgrounds, ensuring robust feature representation of vessel morphology via the background suppression mechanism of the DSAM module.

Low-contrast sea fog scenario (Figure 24c): Under low-contrast conditions caused by dense sea fog, the baseline model is easily overwhelmed by fog-shrouded backgrounds, with weak and discontinuous activation for vessel targets. The proposed model maintains strong and stable feature activation for all ships even in foggy environments, with high-activation regions tightly aligned with vessel contours. This verifies that the proposed model can effectively extract discriminative features of targets from low signal-to-noise ratio scenes, enhancing detection robustness in harsh weather.

Dense coastal vessel scenario (Figure 24d): In scenes with multiple vessels densely clustered along the coastline, the baseline model exhibits noticeable feature response fusion between adjacent targets. It fails to distinguish inter-target gaps, resulting in blurred activation boundaries that hinder subsequent bounding box regression and multi-target counting. Conversely, the proposed model generates balanced and clearly demarcated feature responses for each vessel. Each target forms an independent high-activation region, effectively mitigating feature crosstalk between adjacent objects and significantly reducing the risk of missed detection and misclassification.

In summary, the heatmap visualization results confirm that the proposed AK-DSAM-YOLOv13 effectively suppresses interference from complex maritime backgrounds, while enhancing feature sensitivity to small-scale, occluded, and low-contrast ship targets. These improvements provide a solid feature-level foundation for precise target localization and robust detection under diverse and complex marine conditions, and are fully consistent with the quantitative experimental results.

**Figure 25:**
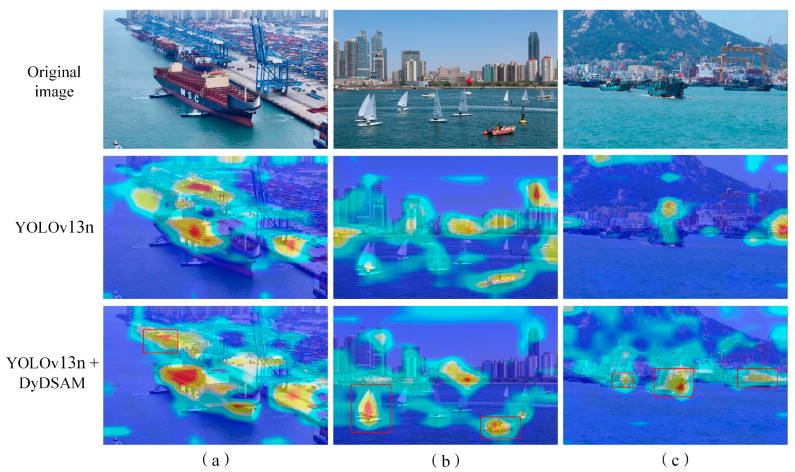
Saliency map visualization of the background clutter suppression effect of the DyDSAM module. Rows from top to bottom: original image, baseline YOLOv13n, and YOLOv13n + DyDSAM. Red and yellow indicate high model attention, blue indicates low attention. Red boxes highlight targets that receive weak or no activation from the baseline but obtain clearly enhanced activation with DyDSAM alone. (**a**) Dense port scene (distant cargo vessel); (**b**) near-shore multi-sailboat scene; (**c**) offshore fishing boat scene.

To visually validate the background suppression capability of DyDSAM, we generated CAM saliency maps comparing feature attention between baseline YOLOv13n and YOLOv13n+DyDSAM on three complex maritime scenes (Figure 25).

The baseline shows severe attention dispersion. In the dense port scene (Figure 25a), responses spread over cranes and container yards, with weak activation for a distant cargo vessel. In the nearshore multi-sailboat scene (Figure 25b), urban backgrounds distract attention, causing extremely weak activation for small sailboats. In the offshore fishing scene (Figure 25c), false responses appear on mountain/coastal backgrounds, while activation for distant small fishing vessels is nearly submerged. This dispersion leads to poor feature representation and high missed-detection risk.

In contrast, YOLOv13n+DyDSAM achieves more focused attention. High-activation regions align better with vessel contours, while non-target regions (buildings, mountains, sea clutter) show noticeably reduced activation. Red boxes highlight the improvement: a distant cargo vessel (Figure 25a), small sailboats (Figure 25b), and distant fishing boats (Figure 25c)—which receive weak or no activation from the baseline—exhibit clearly enhanced activation with DyDSAM alone. This confirms that DyDSAM effectively filters background interference and improves ship feature representation.

These saliency map results are consistent with the quantitative ablation experiments, providing direct evidence for the background suppression effectiveness of DyDSAM.

### 3.5. Error Analysis and Model Limitations

**Figure 26:**
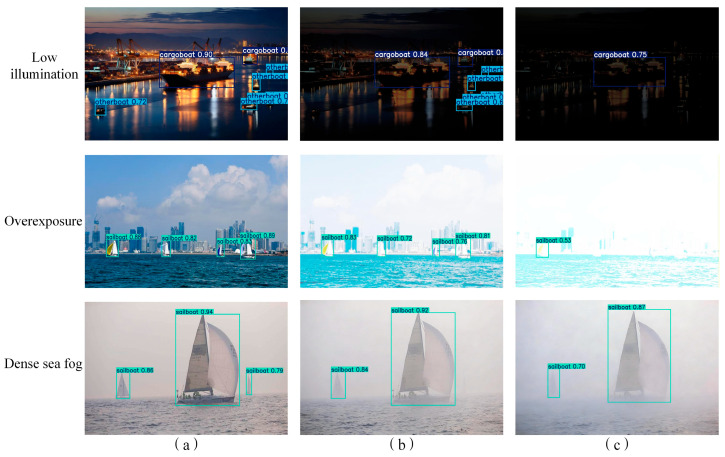
Representative failure cases of AK-DSAM-YOLOv13 on the CM-Ships dataset under extreme conditions. (**a**) Low illumination, (**b**) overexposure, (**c**) dense sea fog.

Despite the superior overall detection performance of the proposed AK-DSAM-YOLOv13 in typical complex marine scenarios, detection failures still occur under a few extreme adverse conditions. Figure 26 illustrates representative failure cases of the proposed model on the CM-Ships dataset, covering three typical extreme adverse scenarios: low illumination, overexposure, and dense sea fog.

In the low illumination scenario (Figure 26a), the contrast between ship targets and the background seawater is drastically reduced, and the images are contaminated by severe dark noise and blurred texture details. Although the model still achieves reliable detection of large cargo vessels with high confidence, the detection confidence of small-scale “other boats” drops significantly (e.g., to 0.72), introducing a non-negligible risk of missed detection. This is a common limitation faced by mainstream lightweight detection models: the shallow features of tiny targets in dark environments are heavily masked by noise, and the lightweight backbone network inevitably sacrifices part of the feature extraction capability under extreme low-light conditions to maintain real-time inference speed.

In overexposed scenes caused by intense sunlight or sea surface specular reflection (Figure 26b), the images suffer from severe high-brightness white noise and color channel distortion. The edges of ship targets are severely blurred, and the target features are easily fused with the overexposed background. With the intensification of overexposure (from left to right in Figure 26b), the detection confidence of the model decreases progressively. When the background is completely overexposed to solid white, the model fails to detect small sailboats in the distance. The core reason for this failure is the loss of discriminative target features under extreme brightness interference. The dynamic receptive field of AKConv may saturate in overexposed regions, making it difficult to capture the subtle contour differences between ship targets and the background.

In the dense sea fog scenario (Figure 26c), light scattering leads to severe image blur and a significant reduction in signal-to-noise ratio. Ship targets, especially tiny distant vessels, are almost completely submerged in the fog, with no valid texture and contour information available for feature extraction. Although large targets can still be detected, the degradation of image quality leads to a reduction in localization accuracy and detection confidence, and the model still struggles to detect tiny distant sailboats under dense fog interference. This is an inherent challenge for visible-light-based ship detection: the down-sampling process of the backbone network further dilutes the already weak features of tiny fog-obscured targets, eventually leading to their misclassification as background.

These typical failure cases demonstrate that extreme lighting conditions (low illumination and overexposure) and severe adverse weather (dense sea fog) remain open challenges for the current visible-light-based marine ship detection system.

## 4. Discussion

In the current landscape of maritime object detection, research has predominantly focused on either accuracy or speed, often at the expense of the other. This study introduces AK-DSAM-YOLOv13, a novel detection framework addressing the core challenges of small-target missed detection, feature discrimination under background clutter, and inaccurate localization in complex marine environments. Our approach integrates three complementary modules: AKC3k2 for adaptive multi-scale feature extraction, DyDSAM for dynamic upsampling with dual-stream attention, and AIoU loss for shape-sensitive regression. Extensive experiments on CM-Ships, SeaShips, and McShips datasets validate its effectiveness, with the proposed model consistently outperforming baseline YOLO models and other state-of-the-art detectors across all benchmarks.

Ablation studies validate each module’s contribution: AKC3k2 improves accuracy while reducing computation; DySample enhances detail retention; DSAM suppresses background interference; AIoU boosts localization precision. Their combined integration yields cumulative gains exceeding individual contributions, indicating effective complementarity.

On the CM-Ships dataset, AK-DSAM-YOLOv13 demonstrates substantial advantages in detection accuracy, providing an effective technical solution for real-time ship detection. On the SeaShips benchmark, its balanced and consistently high-performance color profile across all vessel categories indicates that the integrated design of AKC3k2, DyDSAM, and AIoU loss effectively optimizes detection robustness for multi-category and multi-scale maritime targets. On the McShips dataset, the proposed method achieves comprehensive and balanced performance gains through integrated optimization of feature extraction, multi-scale fusion, and loss function formulation, validating its efficacy and superior generalization capability. Furthermore, as shown in Figure 19, AK-DSAM-YOLOv13 lies on the optimal Pareto frontier of the accuracy-efficiency trade-off, demonstrating an optimal equilibrium between discriminative performance and computational efficiency, making it exceptionally well-suited for real-time maritime surveillance under stringent hardware constraints.

While robust, our model has limitations. Fishing boat and other boat detection lags due to high intra-class variability. Extreme weather robustness could be improved through multi-modal sensor fusion (infrared, radar). Computational costs, though minimized, warrant further optimization for edge deployment. The failure case analysis in Section 3.5 further reveals that extreme lighting conditions (low illumination and overexposure) and severe weather (e.g., dense fog) remain challenging for the current visible-light-based system. These limitations are not unique to our approach but are inherent to optical detection under adverse conditions. Future work will focus on video-based temporal modeling, integration with image restoration algorithms (e.g., contrast enhancement, dehazing), adaptation of infrared-specific methods to RGB inputs, and multi-modal sensor fusion to address these limitations. Despite these considerations, our study provides insights for maritime surveillance and USV applications and may serve as a reference for future research.

## 5. Conclusions

This paper addresses the pivotal challenge of detecting multi-type maritime targets within complex sea environments by proposing a robust detection algorithm, designated as AK-DSAM-YOLOv13. Built upon the YOLOv13n framework, the proposed method realizes a coordinated innovation across three dimensions: feature extraction, multi-scale fusion, and loss function formulation. First, an AKC3k2 module is constructed by leveraging Alterable Kernel Convolution (AKConv) to bolster the model’s adaptive feature extraction capability for multi-scale vessels through dynamic receptive field modulation. Second, a DyDSAM architecture is developed, which integrates the DySample dynamic up-sampling operator with a Dual-Stream Attention Module (DSAM); this configuration optimizes the feature fusion pathway to effectively suppress interference from heterogeneous backgrounds. Third, the Accuracy-Intersection-over-Union (AIoU) loss function is introduced to jointly optimize the intersection area, center distance, and aspect ratio, thereby significantly enhancing the spatial localization precision for dense and small-scale targets. Extensive experimental results on the self-built CM-Ships dataset and the public SeaShips and McShips benchmarks demonstrate that AK-DSAM-YOLOv13 consistently outperforms baseline models and state-of-the-art YOLO-series algorithms across key metrics—including Precision, Recall, and mean Average Precision (mAP)—while maintaining a favorable efficiency trade-off: with 3.84 M parameters and 6.8 GFLOPs, AK-DSAM-YOLOv13 achieves real-time inference at 3.2 ms per image. Compared to YOLOv13n (3.69 M/6.5 GFLOPs, 2.4 ms) and YOLOv8n (3.11 M/10.6 GFLOPs, 3.4 ms), our model offers a competitive efficiency profile, demonstrating that the accuracy improvements do not come at the cost of computational performance. Ablation studies and Grad-CAM++ visual analyses further validate the contribution of each individual module, as well as the model’s superior feature-saliency focusing and environmental robustness under challenging conditions. This study provides a robust technical framework for intelligent visual monitoring in complex marine environments. Future research will prioritize model lightweighting for edge computing (e.g., quantization and pruning), explore the integration of multimodal data (infrared, SAR) for all-weather surveillance, and investigate adaptive image preprocessing techniques (e.g., contrast enhancement, dehazing) to further enhance robustness under extreme conditions.

## Figures and Tables

**Table 1:** Quantitative comparison of core characteristics between AKC3k2 and DS-C3k2 modules.

Aspect	DS-C3k2	AKC3k2	Improvement
Module Structure	Standard Bottleneck blocks; sequential depthwise + pointwise convolution; no adaptive feature adjustment.	AK-Bottleneck blocks; retains parallel branches; introduces offset prediction branch.	Replaces Bottleneck with AK-Bottleneck; adds adaptive adjustment capability.
Convolution Operation	Depthwise separable convolution; fixed kernel shape and size; rigid sampling grid.	Alterable kernel convolution; dynamically adjustable sampling points; content-adaptive offsets.	Learnable sampling points; better alignment with target regions.
Receptive Field	Fixed (default 5 × 5).	Dynamically adaptive.	Flexible coverage based on vessel scale.
Parameter Count (per module)	128.6 k	135.2 k	+5.1%
Computational Cost (per module, GFLOPs)	0.82	0.85	+3.7%

**Table 2:** Dataset composition and source distribution.

Data Source	Number of Images	Proportion (%)	Main Characteristics
Public Datasets	2553	41.4	Mostly top-view or standard side-view angles, relatively clean backgrounds, uniform target scale distribution.
Web Crawling	1352	21.9	Diverse viewpoints, various weather conditions (fog, rain, clear), significant variations in target scale and posture.
Field Collection	2259	36.7	Emphasis on complex sea conditions (waves, fog), nearshore/island backgrounds, small-scale targets, and partial occlusion scenarios.
Total	6164	100	

**Table 3:** Category distribution across training, validation, and test splits.

Categories	ES	OS	CB	SB	PS	Ya	FB	OB	Total
Train	582	599	649	646	633	610	628	586	4933
Val	72	75	81	80	79	76	78	73	614
Test	73	75	81	81	79	76	79	73	614
Total	727	749	811	807	791	762	785	732	6164

Abbreviations: ES (Engineering ships), OS (Official ships), CB (Cargo boats), SB (Sailboats), PS (Passenger ships), Ya (Yachts), FB (Fishing boats), OB (Other boats).

**Table 4:** Number of images of each ship category.

Warship			Civilian Ship		
Category	Images	Percentage	Category	Images	Percentage
Aircraft Carrier	906	0.1139	Containership	1208	0.1351
Auxiliary Ship	926	0.1164	Fishingboat	1660	0.1856
Landing Ship	340	0.0428	Passengership	1219	0.1363
Destroyer	4355	0.5476	Sailboat	2444	0.2733
Submarine	1175	0.1477	Speedboat	1087	0.1216
Missile Boat	251	0.0316	Tugboat	611	0.0683
Total	7953	1	Supportship	713	0.0797
			Total	8942	1

**Table 5:** Model Parameters.

Name	Version/Model	Name	Version/Model
Size	640 × 640	Optimizer	SGD
learning rate	0.01	worker	16
momentum	0.937	batchsize	32
weight_decay	0.0005	epoch	200

**Table 6:** Detection accuracy by target category.

Type	SB	ES	OS	CB	FB	PS	YA	OB	mAP50	Times/ms
AP	0.984	0.958	0.981	0.805	0.756	0.833	0.920	0.779	0.877	3.2 ms

Abbreviations: ES (Engineering ships), OS (Official ships), CB (Cargo boats), SB (Sailboats), PS (Passenger ships), YA (Yachts), FB (Fishing boats), OB (Other boats).

**Table 7:** Comparison experiments based on CM-Ships.

Model	P (%)	R (%)	mAP50 (%)	mAP50:95 (%)	Params (×10^6^)	GFLOPs	Times (ms)
YOLOv8n	84.5 ± 0.2	78.8 ± 0.2	85.1 ± 0.3	65.2 ± 0.2	3.11	10.6	3.4 ± 0.1
YOLOv9n	82.8 ± 0.3	76.8 ± 0.4	83.4 ± 0.2	63.6 ± 0.3	3.70	11.8	3.8 ± 0.1
YOLOv10n	85.8 ± 0.2	78.6 ± 0.1	84.8 ± 0.4	65.7 ± 0.1	2.91	7.1	2.3 ± 0.1
YOLOv11n	86.4 ± 0.2	78.6 ± 0.2	85.4 ± 0.2	65.9 ± 0.3	3.27	6.6	2.5 ± 0.1
YOLOv12n	86.2 ± 0.4	78.5 ± 0.2	85.5 ± 0.1	66.0 ± 0.2	3.80	7.2	2.7 ± 0.1
YOLOv13n	86.7 ± 0.2	78.8 ± 0.1	86.4 ± 0.2 ^1^	66.1 ± 0.3	3.69	6.5	2.4 ± 0.1
AK-DSAM-YOLOv13 (Ours)	**88.6 ± 0.2**	**80.7 ± 0.2**	**87.7 ± 0.2 ^2^**	**67.6 ± 0.2**	**3.84**	**6.8**	**3.2 ± 0.1**

Note: All values are mean ± standard deviation over three independent runs with different random seeds. ^1^ 95% confidence interval for mAP50: [86.1%, 86.8%]; ^2^ 95% confidence interval for mAP50: [87.2%, 88.2%]. Non-overlapping CIs confirm statistical significance (*p* < 0.05, paired *t*-test).

**Table 8:** Comparison experiments based on SeaShips.

Model	P (%)	R (%)	mAP50 (%)	mAP50:95 (%)	Params (×10^6^)	GFLOPs	Times (ms)
Faster R-CNN	84.7 ± 0.2	75.7 ± 0.1	88.5 ± 0.1	69.3 ± 0.2	34.52	42.1	49.8 ± 0.1
Libra R-CNN	81.6 ± 0.2	71.4 ± 0.2	85.3 ± 0.2	65.7 ± 0.1	31.75	43.0	43.7 ± 0.1
SSD	86.4 ± 0.2	76.5 ± 0.1	87.4 ± 0.1	67.8 ± 0.1	12.26	29.9	11.1 ± 0.1
YOLOv8n	92.3 ± 0.2	87.6 ± 0.1	92.7 ± 0.1	72.4 ± 0.1	3.11	10.6	3.4 ± 0.1
YOLOv9n	91.9 ± 0.1	86.4 ± 0.1	92.2 ± 0.1	70.4 ± 0.1	3.70	11.8	3.8 ± 0.1
YOLOv10n	92.8 ± 0.1	86.3 ± 0.1	93.4 ± 0.2	73.4 ± 0.1	2.91	7.1	2.3 ± 0.1
YOLOv11n	93.1 ± 0.1	87.6 ± 0.2	93.1 ± 0.3	73.7 ± 0.2	3.27	6.6	2.5 ± 0.1
YOLOv12n	92.7 ± 0.2	88.4 ± 0.1	93.6 ± 0.1	73.3 ± 0.1	3.80	7.2	2.7 ± 0.1
YOLOv13n	93.4 ± 0.1	88.7 ± 0.1	93.6 ± 0.1 ^1^	73.9 ± 0.2	3.69	6.5	2.4 ± 0.1
FCOS	79.4 ± 0.2	67.8 ± 0.1	83.6 ± 0.1	70.6 ± 0.1	24.34	57.6	42.8 ± 0.1
Deformable DETR	92.3 ± 0.2	84.6 ± 0.2	92.7 ± 0.2	71.7 ± 0.2	22.45	46.7	25.2 ± 0.1
DINO	93.6 ± 0.2	86.2 ± 0.1	93.5 ± 0.2	72.4 ± 0.1	28.71	50.2	31.8 ± 0.1
AK-DSAM-YOLOv13 (Ours)	**94.6 ± 0.1**	**91.4 ± 0.2**	**95.1 ± 0.1 ^2^**	**76.3 ± 0.1**	**3.84**	**6.8**	**3.2 ± 0.1**

Note: All values are mean ± standard deviation over three independent runs. ^1^ 95% confidence interval for mAP50: [93.4%, 93.8%]; ^2^ 95% confidence interval for mAP50: [94.9%, 95.3%]. Non-overlapping CIs confirm statistical significance (*p* < 0.05).

**Table 9:** Comparison experiments based on McShips.

Model	P (%)	R (%)	mAP50 (%)	mAP50:95 (%)	Params (×10^6^)	GFLOPs	Times (ms)
Faster R-CNN	82.4 ± 0.1	74.8 ± 0.1	87.1 ± 0.2	67.3 ± 0.1	34.52	42.1	49.8 ± 0.1
Libra R-CNN	80.8 ± 0.1	70.2 ± 0.1	84.5 ± 0.2	62.2 ± 0.1	31.75	43.0	43.7 ± 0.1
SSD	85.4 ± 0.1	77.3 ± 0.1	88.2 ± 0.2	65.5 ± 0.1	12.26	29.9	11.1 ± 0.1
YOLOv8n	89.8 ± 0.2	86.7 ± 0.3	92.4 ± 0.1	71.0 ± 0.1	3.11	10.6	3.4 ± 0.1
YOLOv9n	88.7 ± 0.2	85.0 ± 0.2	91.8 ± 0.1	69.2 ± 0.1	3.70	11.8	3.8 ± 0.1
YOLOv10n	89.9 ± 0.1	86.1 ± 0.1	92.5 ± 0.1	70.5 ± 0.1	2.91	7.1	2.3 ± 0.1
YOLOv11n	90.7 ± 0.1	87.0 ± 0.1	92.7 ± 0.2	70.7 ± 0.1	3.27	6.6	2.5 ± 0.1
YOLOv12n	90.4 ± 0.2	87.7 ± 0.2	92.3 ± 0.2	70.3 ± 0.1	3.80	7.2	2.7 ± 0.1
YOLOv13n	91.0 ± 0.2	88.3 ± 0.2	92.6 ± 0.2 ^1^	71.4 ± 0.2	3.69	6.5	2.4 ± 0.1
FCOS	83.8 ± 0.3	66.7 ± 0.2	82.2 ± 0.2	61.3 ± 0.1	24.34	57.6	42.8 ± 0.1
Deformable DETR	91.3 ± 0.2	85.0 ± 0.1	92.0 ± 0.2	69.7 ± 0.2	22.45	46.7	25.2 ± 0.1
DINO	90.6 ± 0.1	85.3 ± 0.1	92.7 ± 0.1	71.0 ± 0.2	28.71	50.2	31.8 ± 0.1
AK-DSAM-YOLOv13 (Ours)	**93.3 ± 0.1**	**89.8 ± 0.1**	**93.8 ± 0.1 ^2^**	**73.3 ± 0.1**	**3.84**	**6.8**	**3.2 ± 0.1**

Note: All values are mean ± standard deviation over three independent runs. ^1^ 95% confidence interval for mAP50: [92.2%, 93.0%]; ^2^ 95% confidence interval for mAP50: [93.6%, 94.0%]. Non-overlapping CIs confirm statistical significance (*p* < 0.05).

**Table 10:** Detection result comparison of ablation experiments.

AKConv	DySample	DSAM	AIoU	P/%	R/%	mAP50/%	mAP50:95/%	Params/10^6^	GFLOPs	Times/ms
×	×	×	×	86.5	78.9	86.3	65.8	3.69	6.5	2.4
√	×	×	×	86.7	78.8	86.5	66.3	3.82	6.5	2.8
×	√	×	×	86.8	79.1	86.6	66.7	3.84	6.6	3.0
×	×	√	×	87.0	79.0	86.7	66.7	3.77	7.0	3.1
×	×	×	√	86.6	79.2	86.4	66.8	3.69	6.5	2.4
√	√	×	×	87.4	79.5	86.6	67.1	3.54	6.5	2.9
√	√	√	×	87.8	79.9	86.8	67.2	3.68	6.9	3.0
√	√	√	√	**88.4**	**80.7**	**87.7**	**67.6**	**3.84**	**6.8**	**3.2**

Note: “×” = without the corresponding module; “√” = with the corresponding module.

**Table 11:** Comparison of different IoU-based loss functions on the CM-Ships dataset.

Loss Function	P/%	R/%	mAP50/%	mAP50:95/%	Times/ms
CIoU (baseline)	87.8	79.9	86.8	67.2	3.0
DIoU	87.9	80.0	86.9	67.3	3.0
GIoU	87.7	79.8	86.7	66.9	3.1
EIoU	88.0	80.2	87.1	67.4	3.1
WIoU	88.1	80.4	87.3	67.5	3.1
AIoU	88.4	80.7	87.7	67.6	3.2

**Table 12:** Comparison of upsampling operators on the CM-Ships dataset.

Upsampling Method	P/%	R/%	mAP50/%	mAP50:95/%	Params/10^6^	GFLOPs	Times/ms
Nearest (Baseline)	88.0	80.2	87.3	67.2	3.69	6.5	3.0
Bilinear	88.1	80.4	87.4	67.3	3.69	6.5	3.1
Bicubic	88.1	80.5	87.4	67.3	3.69	6.5	3.1
CARAFE	88.5	80.6	87.6	67.4	3.90	7.2	3.5
DySample	88.4	80.7	87.7	67.6	3.84	6.8	3.2

**Table 13:** Performance comparison between AKC3k2 and DS-C3k2 modules on the CM-Ships small-scale subset.

Module	P (%)	R (%)	mAP50 (%)	mAP50:95 (%)	Inference Time (ms/img)
DS-C3k2	78.2	72.5	76.8	52.3	2.4
AKC3k2	82.5	77.8	81.2	56.7	2.8

Note: This experiment isolates the effect of replacing DS-C3k2 with AKC3k2 on the small-scale subset; all other network components and training settings are identical.

## Data Availability

The original contributions presented in this study are included in the article. Further inquiries can be directed to the corresponding author.
